# Using a novel structure/function approach to select diverse swine major histocompatibility complex 1 alleles to predict epitopes for vaccine development

**DOI:** 10.1093/bioinformatics/btad590

**Published:** 2023-09-22

**Authors:** Zahed Khatooni, Navid Teymourian, Heather L Wilson

**Affiliations:** Vaccine and Infectious Disease Organization (VIDO), University of Saskatchewan, Saskatoon, SK S7N 5E3, Canada; Department of Computer Science, University of Kurdistan, Sanandaj, Iran; Department of Veterinary Microbiology, Western College of Veterinary Medicine, University of Saskatchewan, Saskatoon, SK S7N 5B4, Canada; Vaccine and Infectious Disease Organization (VIDO), University of Saskatchewan, Saskatoon, SK S7N 5E3, Canada; Department of Computer Science, University of Kurdistan, Sanandaj, Iran; Department of Veterinary Microbiology, Western College of Veterinary Medicine, University of Saskatchewan, Saskatoon, SK S7N 5B4, Canada; Vaccinology & Immunotherapeutics Program, School of Public Health, University of Saskatchewan, Saskatoon, SK S7N 5B4, Canada

## Abstract

**Motivation:**

Swine leukocyte antigens (SLAs) (i.e. swine major histocompatibility complex proteins) conduct a fundamental role in swine immunity. To generate a protective vaccine across an outbred species, such as pigs, it is critical that epitopes that bind to diverse SLA alleles are used in the vaccine development process. We introduced a new strategy for epitope prediction.

**Results:**

We employed molecular dynamics simulation to identify key amino acids for interactions with epitopes. We developed an algorithm wherein each SLA-1 is compared to a crystalized reference allele with unique weighting for non-conserved amino acids based on R group and position. We then performed homology modeling and electrostatic contact mapping to visualize how relatively small changes in sequences impacted the charge distribution in the binding site. We selected eight diverse SLA-1 alleles and performed homology modeling followed, by protein–peptide docking and binding affinity analyses, to identify porcine reproductive and respiratory syndrome virus matrix protein epitopes that bind with high affinity to these alleles. We also performed docking analysis on the epitopes identified as strong binders using NetMHCpan 4.1. Epitopes predicted to bind to our eight SLA-1 alleles had equivalent or higher energetic interactions than those predicted to bind to the NetMHCpan 4.1 allele repertoire. This approach of selecting diverse SLA-1 alleles, followed by homology modeling, and docking simulations, can be used as a novel strategy for epitope prediction that complements other available tools and is especially useful when available tools do not offer a prediction for SLAs/major histocompatibility complex.

**Availability and implementation:**

The data underlying this article are available in the online Supplementary Material.

## 1 Introduction

Human food security depends on sufficient protein derived from the swine industry. Several infectious agents, such as porcine reproductive and respiratory syndrome virus (PRRSV), circovirus, *Lawsonia intracellularis*, rotavirus, etc., affect pig reproduction or the health of newborn and growing pigs (Lammers *et al.* 2009, Neeteson-van Nieuwenhoven *et al.* 2013, Montaner-Tarbes *et al.* 2019, Kezia *et al.* 2021). Developing safe and effective vaccines against infectious diseases that are highly effective across pig populations can be improved with greater comprehensive knowledge of swine immunology.

Swine leukocyte antigens (SLAs) are major histocompatibility complex (MHC) proteins; they share homology with the human MHC or HLAs (Lunney *et al.* 2009, VanderWaal and Deen 2018). SLAs are located on Chromosome 7 and organized into Classes I, II, and III (Chardon *et al.* 1999, Renard *et al.* 2006, Hammer *et al.* 2020). There are approximately >150 loci and 120 functional (encode proteins) genes for SLA alleles (Hammer *et al.* 2020). According to the Immuno Polymorphism Database (IPD) (https://www.ebi.ac.uk/ipd/mhc/group/SLA/), functional class 1a SLA genes consist of 100 SLA-1 alleles, 105 SLA-2 alleles, and 47 SLA-3 alleles (subject to change). Functional class 1b SLA-1 genes consist of 10 SLA-6 alleles for a total of 262 Class-I alleles (Maccari *et al.* 2020, Robinson *et al.* 2020). To date, there are limited crystal structures of SLA-1 alleles, including SLA-1*04:01, SLA-1*13:01, SLA-1*1502, and SLA-1*14:02 (Zhang *et al.* 2011, Ning *et al.* 2020, Wei *et al.* 2021, Pan *et al*. 2020) (https://www.rcsb.org/).

The means to identify SLAs and the epitopes within infectious agents are still among the major unsolved challenges (Purcell *et al.* 2007, Fan *et al.* 2016, 2018). Because only some SLA alleles are found in high frequency within multiple pig populations, it may seem reasonable to select epitopes that bind to these well-represented SLA peptide-binding regions for vaccine development, but this approach may lead to increased chances of immune escape over time (Ning *et al.* 2020). Alternatively, by including diverse SLAs for vaccine development, one can select epitopes from an infectious agent to include in vaccines that protect pigs across the population with reduced risk of immune escape.

**Figure 1.**
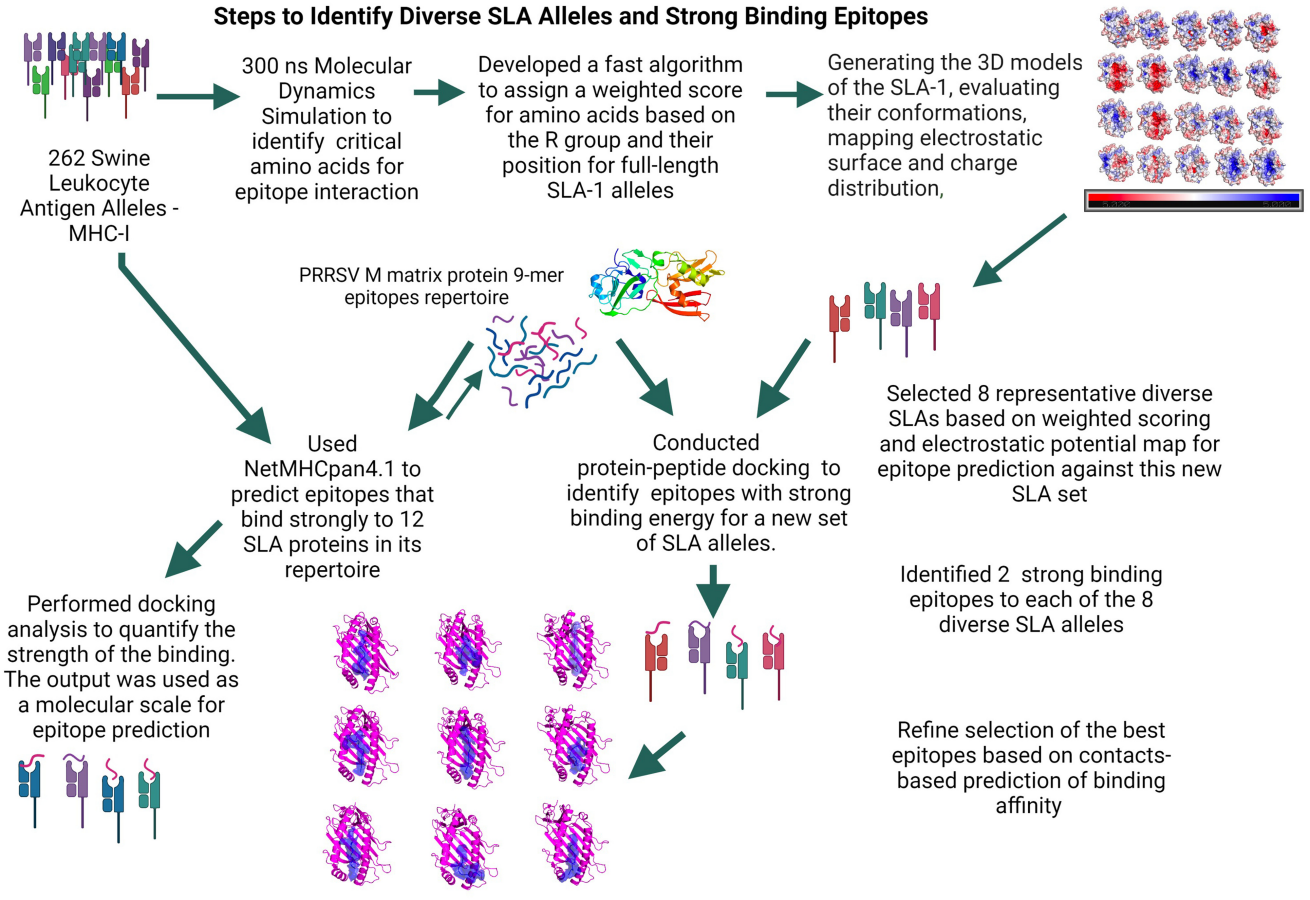
Schematic of our *in silico* approach for SLA classification and T-cell epitope prediction. MDS was used to identify the amino acids in the SLA-1 that are critical for interaction with the epitope, using the crystalized epitope bound to SLA-1*04:01 as the reference allele. We designed an algorithm to compare SLA-1 alleles to the reference allele, taking into consideration the similarity of the amino acid sequence but also the charge, size, and position of the non-conserved amino acids. Assessment of the conformations and electrostatic surface maps of the whole SLAs and the peptide-binding pocket was used to identify eight diverse SLA-1 selected to represent SLA-1. NetMHCpan 4.1 tool was used to identify the PRRSV M protein epitopes. We performed docking analysis to quantify the strength of binding of the epitopes-SLAs identified using NetMHCpan 4.1 and compared them to the epitopes that were selected to bind to our eight diverse SLA-1 alleles (these epitopes were the weak epitope against 12 SLAs in the tool list). We selected the top 2 epitopes that bound to each of the diverse SLAs to use for vaccine development. Schematic created using BioRender.com (2022).

Here, we investigated a new approach to assess SLA allele similarities based on the amino acid position, charge, and size of the non-conserved amino acids. Single residue modifications play a critical role in protein structure and function (Temmam *et al.* 2022, Zhang *et al.* 2022). In our algorithm, the crystalized and high-frequency allele, SLA-1*04:01, was used as a reference, and all other SLA-1 alleles are compared to it. We performed a molecular dynamics simulation (MDS) to confirm the amino acids that play a role in the stabilization of the molecule and the interaction with the peptide (Fig. 1). Each non-conserved amino acid was assigned a weighted score considering its position (i.e. whether they play a role in epitope binding or stability) and the differences in the amino acid R group size and charge. Next, we performed surface charge mapping, which revealed that relatively small differences in amino acid sequences affected the charge distribution of the peptide-binding domain. We selected eight SLA-1 alleles, some of which are highly, moderately, or poorly similar to the SLA-1*04:01 allele based on critical amino acid interaction (CAAI) scoring and their surface charge distribution. As a representative of available epitope prediction tools and the accuracy of their output, we used NetMHCpan 4.1 to identify PRRSV matrix (M) protein epitopes (strong and weak) that bind 12 SLA-1 alleles in its repertoire and evaluate whether its weak predicted epitopes can be strong binders of our eight SLA-1 that are not part of its repertoire (up to now). Finally, we performed homology modeling followed by protein–peptide docking analysis to identify the M protein epitopes that bind strongly to our eight diverse SLA-1 alleles. When we performed docking analysis to compare the strength of the binding energies across the two approaches, some M protein epitopes had stronger interactions with the eight SLA-1 alleles than they did with the epitopes predicted as strong binders to the 12 SLA-1. SLA selection based on sequence, in addition to well-established homology modeling and docking simulations, can be used as a new means for epitope prediction, as a complementary of well-recognized tools (i.e. NetMHCpan) or an independent for newly discovered MHCs (Fig. 1).

## 2 Materials and methods 

### 2.1 Molecular dynamics simulation

**Figure 2.**
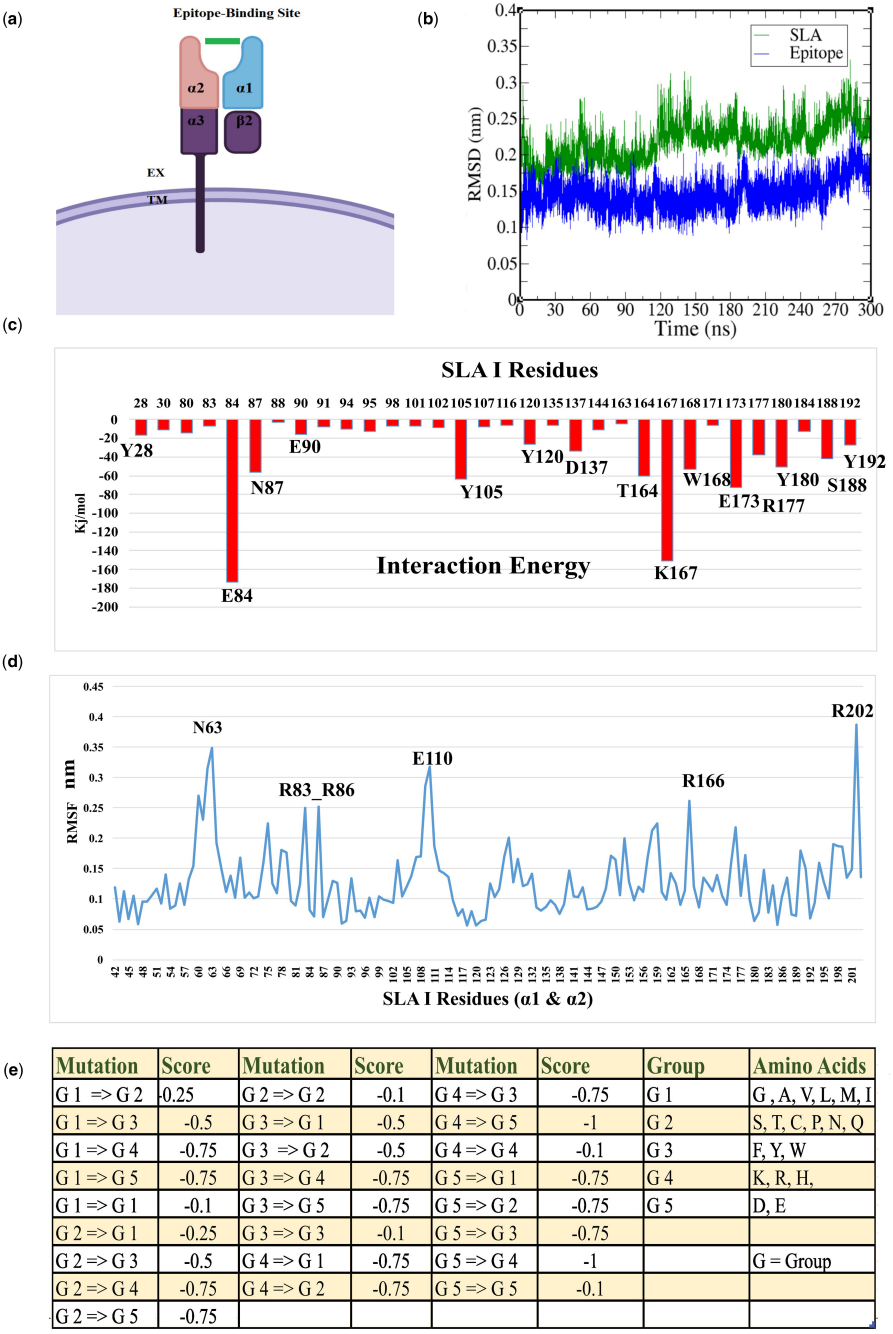
MDS results and scoring function. (a) Schematic of the standard structure of MHC-1 domains adapted from “MHC Class I,” by BioRender.com (2022). The TM region and the extracellular region of the protein are noted. The epitope binding domain is comprised of α1 and α2 that connected to each other, aa 22–203. The schematic of epitope is shown in green (b) RMSD, a measure of the stability, was calculated on the backbone atoms for the SLA-1 (SLA-1*04:01:01 PDB: 3qq3) and its crystalized epitope in green and blue, respectively. (c) The IE (sum of Coulomb and van der Waals) from the last 50 ns (250–300 ns) of the MDS between the residues of SLA-1 and its crystalized epitope. Only residues with IE more than −5 kJ/mol are shown. (d) The RMS fluctuation (nm) for the last 50 ns of the MD simulation of reference SLA-1. The SLA-1 residues are shown in a blue graph, and the most flexible residues are indicated using the single amino acid code. (e) Developed scoring functions and group classifications for SLA-1 alleles. The amino acids in each group are indicated in the last column.

MDS was conducted on SLA-1 for 300 ns using the GROMACS 2021.1 (https://www.gromacs.org/) (Fig. 2b–d). The all atoms CHARMM36 were used as a force field (Best *et al.* 2012, Abraham *et al.* 2015, Huang *et al.* 2017, Van Der Spoel *et al.* 2005). The cubic shape simulation box with 1.0 nm distance between its edge and SLA–epitope complex filled with TIP3P water models. The system was neutralized by adding Na+ atoms, and atomic coordinates were optimized by employing the steepest descent minimization algorithm. Fluctuations of temperature (at 300 K) and pressure (1 bar) were avoided by applying the modified Berendsen thermostat (V-rescale) with the time constant τ_*t* = 0.1 ps and Parrinello–Rahman pressure coupling at compressibility of 4.5e-5 and τ_ *p* = 2 ps (Parrinello and Rahman 1981, Berendsen *et al.* 1984, Bussi *et al.* 2007). The LINCS algorithm was used as the constraint scheme for bond lengths (Hess *et al.* 1997). The time step for simulation was set to 2 fs, and “md” integrator was employed to integrate Newton’s equations of motion. The van der Waals and electrostatic interactions were treated by Lennard Jones and Particle Mesh Ewald with assigned 1.2 nm cut-off, respectively (Darden *et al.* 1993). Before the production MD for 300 ns, the Position Restraint (NVT ensemble) and NPT simulation (NPT ensemble) were performed for 1 and 5 ns, respectively, while using the same 2 fs time step. Tools and software for visualization or generating graphs included VMD (https://www.ks.uiuc.edu/Research/vmd/), PyMol (https://pymol.org/2/), and Grace (https://plasma-gate.weizmann.ac.il/Grace/).

### 2.2 Prediction of transmembrane helices within sequences of SLA-1 alleles

We submitted the SLA-1 alleles to TMHMM - 2.0 (https://services.healthtech.dtu.dk/service.php?TMHMM-2.0) using the default parameters to predict the residues that likely comprise the transmembrane (TM) helices.

### 2.3 Algorithm to automate the SLA sequence diversity calculation

To rank and scores the SLA alleles, an algorithm (Supplementary Data, Pages 1–6) was designed that took into account each amino acid position and whether its R group was similar in size and charge relative to the corresponding amino acid in the SLA-1*04:01 reference allele. This information was used to generate a score for each non-conserved amino acid and then the full-length protein for SLA-1 alleles. After evaluation, it generates a new sheet including all amino acids, their mutations, and scores for each mutated allele one by one (Lutellier *et al.* 2018, Teymourian *et al.* 2022).

### 2.4 Homology modelling of the SLA-1 proteins to predict their 3D conformations and electrostatic contact maps

**Figure 3.**
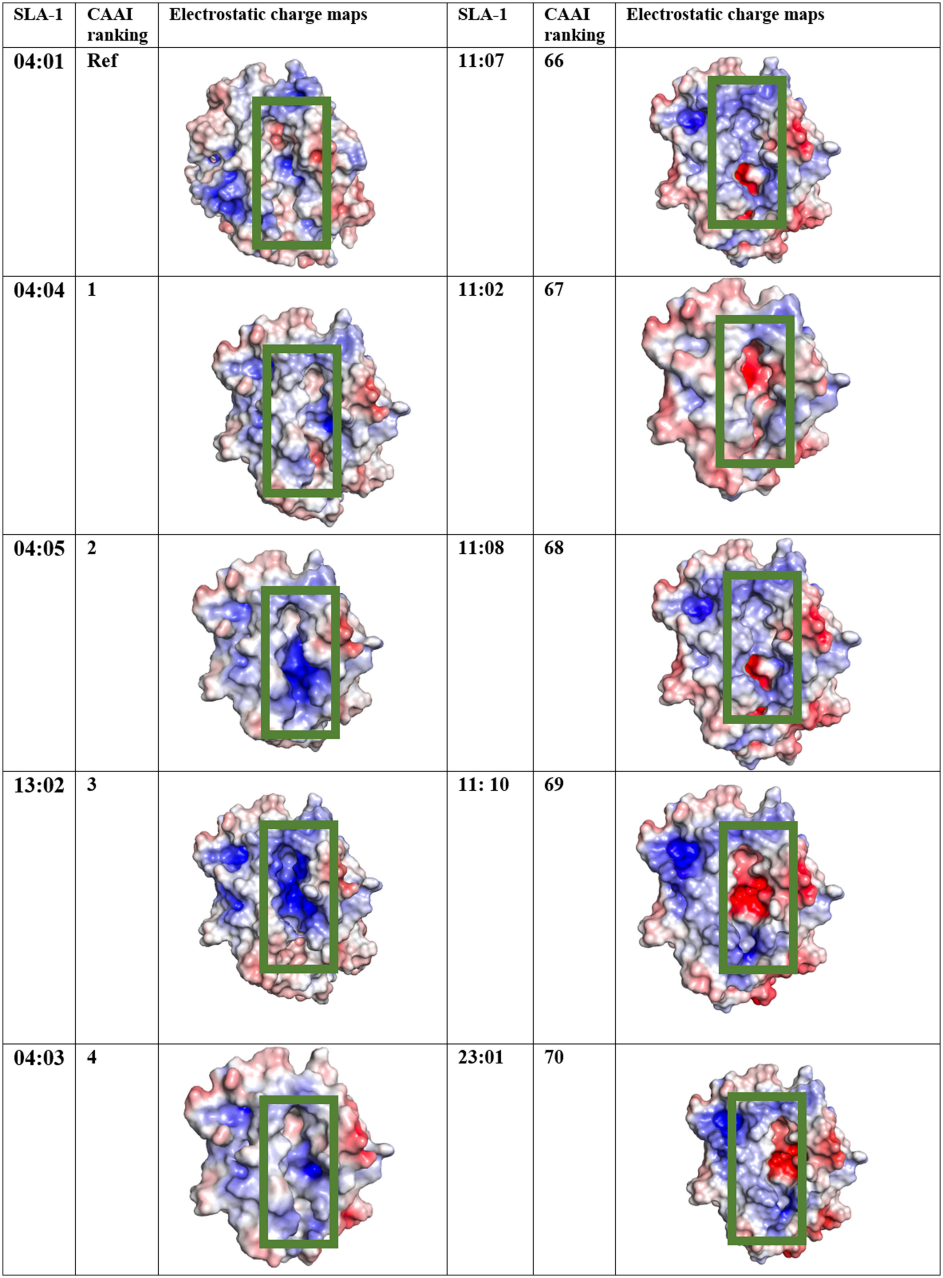
Electrostatic contact maps of the SLA-1 with most and least sequence homology to the reference allele. ^a^Allele names were identified using Immuno polymorphism Database (https://www.ebi.ac.uk/ipd/mhc/group/SLA/). ^b^For generating electrostatic maps, the PDB format was changed to PQR by APBS-PDB2PQR software suite. Next, the SLA-1 alleles were submitted to the APBS electrostatics plugin in PyMol to quantify the electrostatic charge throughout the protein. The charge distribution of the binding site (in general, the whole SLA) can be strongly positive or negative, which displays with blue and red, respectively, in this evaluation. Deep blue or red are indications of strong positive or negative charges, and loss of color shows the neutralized region is dominant. The comparison of the color and quantification based on the explained methods can be seen in Supplementary Fig. S3 for SLA-1 02:01.

The 3D structure of SLA-1 alleles and selected epitopes were modeled and evaluated through SWISS-MODEL (http://swissmodel.expasy.org/), HEPDOCK, and CABS-dock server (Guex *et al.* 2009, Song *et al.* 2013, Kurcinski *et al.* 2015, Waterhouse *et al.* 2018, Zhou *et al.* 2018, Studer *et al.* 2020). To ensure all generated structures were relaxed and their residues are positioned in terms of allowed ϕ and ψ, the Ramachandran plots for all residues were calculated (Supplementary Figs S4 and S5). Results showed that for all SLA-1 models, >92% of their residues were found in the allowed regions. To generate electrostatic maps, the PDB format was changed to PQR (primary input format for biomolecular structure in APBS package) by submitting the PDB coordinates to APBS-PDB2PQR software suite (https://server.poissonboltzmann.org/). Next, the SLA-1 alleles were submitted to the APBS electrostatics (Adaptive Poisson–Boltzmann Solver) plugin in PyMol to quantify the electrostatic charge throughout the protein (see Fig. 3 and Supplementary Figs S1–S3).

### 2.5 Epitope prediction based on analysis from full-length PRRSV M protein

We submitted the PRRSV M protein sequence (GenBank: ABL60903.1) to NetMHCpan 4.1 to generate a list of strong (predicted) epitopes that bind to the 12 SLA-1 alleles available through this tool(Nielsen *et al.* 2007, Reynisson *et al.* 2020) (strong binders) using its default criteria. These epitopes were docked, and their binding energy was used as a scale for the molecular docking of unknown epitopes. The epitopes predicted to be weak binders to all or some of 12- mentioned SLA-1 were docked to eight SLA-1, which are not included in the available epitope mapping tools (Supplementary Table S4).

### 2.6 Protein–peptide docking and evaluation of protein–peptide binding

Molecular docking was performed with the selected SLA-1 alleles and (strong and weak) predicted epitopes generated through NetMHCPan 4.1 from PRRSV M protein. Docking was performed using HADDOCK (https://bianca.science.uu.nl/haddock2.4/) (van Zundert *et al.* 2016, Honorato *et al.* 2021). The structures of SLA-1 alleles were generated through SWISS-MODEL. We then manually curated them to ensure they had accurate and validated docking poses. Then, 16 predicted epitopes were subjected to PRODIGY (https://bianca.science.uu.nl/prodigy/) to assess peptide–protein binding affinity (Vangone and Bonvin 2015, Elez *et al.* 2018). The antigenicity of the selected epitopes was evaluated by submitting them to VaxiJen (Doytchinova and Flower 2007) tool using default parameters.

## 3 Results

### 3.1 MDS used to identify critical amino acids for epitope binding

SLA-1 proteins are organized into [±3 amino acids; a leader sequence from 1 to 21 amino acids, α1 (Residues 22–112), α2 (Residues 113–203), α3 (Residues 204–295), extracellular M (296–303 amino acids), TM helix (Residues 304–326), and cytoplasm domain (Residues 327–361)]. SLA-1*04:01:01 is a well-studied and high-frequency SLA-1 allele (Zhang *et al.* 2011), which we selected as our reference allele. Like all full-length SLA-1 alleles, its peptide-binding site is composed of α1 and α2 helices that are connected to an Ig-like α3 helix. The α3 helix is linked to the TM helix through a short extracellular region, and a cytosolic C-terminal domain extends from the TM helix (Zhang *et al.* 2011, Hammer *et al.* 2020, Ning *et al.* 2020). The β2 microglobulin is not involved in peptide interaction directly but may play a role in the stability of the whole SLA-1: epitope complex (Fig. 2a depicts a schematic of the MHC-I protein).

We performed MDS on the crystal structure of SLA-1*04:01:01 to identify the amino acids that impact SLA–epitope binding and stability (Fig. 2b). This MDS was performed for 300 ns and sampled several million conformations of the SLA-1*04:01:01 crystalized allele and its bound epitope. The van der Waals and Coulomb energies between the epitopes and SLA-1 residues were evaluated for the last 50 ns of the MDS; their sums give the interaction energy (IE) (Fig. 2c). The IE analysis shows that the major sites of interactions between the epitope and SLA-1 *04:01:01 were within the ∼180 amino acids that comprise the α1 and α2 helices, which shows agreement with other studies (Ning *et al.* 2020). Furthermore, several charged and aromatic residues within the α1 and α2 peptide-binding domains have strong (−40 kJ/mol or higher) IE, indicating they are critical amino acids responsible for stabilizing the epitope-SLA-1*04:01:01 interaction. These residues include Glu84, Lys167, Glu173, Tyr105, Thr164, Asn87, Trp168, and Try180 with −173.7, −150.6, −72.5, −63.9, −60.2, −56.2, −53.1, and −51.3 kJ/mol IE, respectively (Fig. 2c). Finally, we assessed the flexibility of each amino acid in 3D space using gmx rmsf. Most residues had <0.22 nm average flexibility, indicating that the reference crystal SLA-1*04:01:01 and its epitope were relatively stable (Fig. 2d). However, some residues within the reference SLA-1*04:01:01 α1 and α2 helices showed structural fluctuation of >0.24 nm, including Arg202 (0.38 nm), Asn63 (0.34 nm), Glu110 (0.31 nm), Arg83 (0.25 nm), Arg86 (0.25 nm), and Arg166 (0.26 nm). This higher flexibility might be in favor or against the positioning of any epitopes depending on the sequence (Fig. 2d).

Performing this MDS on a crystalized SLA-1 and its bound epitope confirmed that the amino acids for epitope binding fall within the α1 and α2 domains. We developed a MATLAB algorithm (Supplementary Data, Pages 1–6) to calculate a score for each amino acid wherein more weight was given to amino acids predicted to impact epitope binding, i.e. amino acids within the α1 and α2 domains. If the amino acid from the allele of interest deviated from the reference allele, it was assigned a score as follows: non-conserved amino acids within the leader, α3 helix, extracellular domain, and cytoplasmic domains were deemed to play a non-critical role in epitope stability, were assigned a score of −0.1. Because amino acids in the TM helix play a role in stability but have no direct interactions with the epitope, non-conserved amino acids in the TM helix were assigned a score of −0.2. Non-conserved amino acids within the α1 and α2 helices were assigned a score of −0.5 as they are part of the peptide-binding groove. Furthermore, we assigned an additional score to each non-conserved amino acid based on the nature of the R group to reflect the impact it will have on SLA–epitope stability (Fig. 2e). For example, amino acids are organized into five categories based on their R groups as follows: Group 1 (non-polar; Gly, Ala, Val, Leu, Met, and Ile), Group 2 (polar; Ser, Thr, Cys, Pro, Asn, and Gln), Group 3 (aromatic; Phe, Tyr, and Trp), Group 4 (positively charged; Lys, Arg, and His), and Group 5 (negatively charged; Asp and Glu) (Nelson 2005). When the amino acid of the reference allele and the allele of interest were different but within the same amino acid group, they were assigned an additional charge of −0.1. If an amino acid from Group 1 was mutated to an amino acid from Group 2 (or *vice versa*), they were assigned an additional score of −0.25 to reflect those changes in R group polarity that will affect epitope stability. Likewise, if an amino acid in Group 3 was substituted with an amino acid from Group 1 or 2 (or *vice versa*), they were assigned an additional charge of −0.5. Any substitution of residues from Group 4 or 5 to any residues of Groups 1, 2, and 3 (or *vice versa*) was assigned a score of −0.75. Finally, if the non-conserved amino acid was in Group 4, but the reference sequence amino acid was in Group 5 (or *vice versa*), the amino acid was assigned an additional score of −1 due to the impact that the different charge will have on the epitope binding (Fig. 2e). Using this method (i.e. the CAAI), the similarities or differences in amino acid sequences between SLA-1 alleles and the reference allele can be quantified. The CAAI score and rank for 70 functional SLA-1 alleles are shown in Table 1.

**Table 1.** CAAI scoring.^a^

Allele accession #	Allele name	Similarity based on sequences		Similarity based on CAAI scoring function	
		Identity score	Rank	Score	Rank
SLA06108	SLA-1*04:04	99.446	1	−0.8	1
SLA06109	SLA-1*04:05	99.169	2	−2.7	2
SLA06132	SLA-1*13:02	97.784	3	−7.4	3
SLA06107	SLA-1*04:03	97.507	6	−7.4	4 #
SLA06131	SLA-1*13:01	97.507	4	−7.95	5 #
SLA08460	SLA-1*17:02	97.23	7	−8.75	6 #
SLA06112	SLA-1*06:02	96.953	8	−9.15	7 #
SLA08440	SLA-1*04:02	97.507	5	−9.35	8 #
SLA06111	SLA-1*06:01	96.953	9	−11.4	9
SLA06103	SLA-1*02:01	94.737	14	−15.4	10 #
SLA06135	SLA-1*17:01	95.568	10	−15.9	11 #
SLA08458	SLA-1*15:02	95.291	11	−17.2	12 #
SLA06104	SLA-1*02:02	94.737	13	−17.2	13
SLA08457	SLA-1*15:01	95.014	12	−17.8	14 #
SLA06105	SLA-1*02:03	94.46	15	−17.8	15
SLA06138	SLA-1*07:03	93.352	19	−20	16 #
SLA06100	SLA-1*01:01	93.629	16	−20.5	17 #
SLA06114	SLA-1*07:02	93.629	17	−21	18 #
SLA06113	SLA-1*07:01	93.352	18	−21.8	19 #
SLA06115	SLA-1*08:01	92.244	24	−22.3	20 #
SLA06116	SLA-1*08:02	92.244	25	−22.6	21 #
SLA08444	SLA-1*08:06	92.244	27	−23.6	22 #
SLA06117	SLA-1*08:12	91.69	36	−23.8	23 #
SLA08449	SLA-1*09:01	92.521	23	−24.1	24 #
SLA08448	SLA-1*08:14	91.967	30	−24.1	25 #
SLA06141	SLA-1*09:02	92.798	22	−24.5	26 #
SLA06140	SLA-1*07:05	91.967	29	−25	27 #
SLA06130	SLA-1*12:02	92.244	26	−25	28 #
SLA06129	SLA-1*12:03	92.798	20	−25.2	29 #
SLA06128	SLA-1*12:01	92.798	21	−25.2	30 #
SLA06119	SLA-1*08:07	91.967	33	−25.7	31 #
SLA08441	SLA-1*07:04	91.413	38	−26	32 #
SLA06136	SLA-1*14:02	91.69	34	−26.4	33 #
SLA06126	SLA-1*11:04	91.967	31	−27.1	34 #
SLA06139	SLA-1*18:01	91.967	28	−27.1	35 #
SLA08446	SLA-1*08:10	90.582	47	−27.3	36 #
SLA09741	SLA-1*08:18	90.859	45	−27.4	37 #
SLA09716	SLA-1*21:03	90.305	52	−27.6	38 #
SLA08456	SLA-1*14:03	91.69	37	−27.6	39 #
SLA09714	SLA-1*08:15	90.859	46	−27.6	40 #
SLA06118	SLA-1*08:13	90.305	50	−27.7	41 #
SLA09719	SLA-1*15:03	91.69	35	−27.9	42 #
SLA08442	SLA-1*07:06	91.136	41	−27.9	43 #
SLA09742	SLA-1*14:04	91.413	39	−28.1	44 #
SLA06120	SLA-1*08:08	90.582	49	−28.4	45 #
SLA06121	SLA-1*08:03	90.582	48	−28.6	46 #
SLA09743	SLA-1*14:05	90.859	42	−28.7	47 #
SLA08454	SLA-1*11:09	91.967	32	−28.7	48 #
SLA06137	SLA-1*14:01	90.859	43	−29	49 #
SLA06122	SLA-1*08:05	90.028	54	−29.2	50 #
SLA08462	SLA-1*20:01	90.859	44	−29.7	51 #
SLA06127	SLA-1*11:06	91.136	40	−30.6	52 #
SLA09713	SLA-1*21:01	89.474	61	−31.4	53 #
SLA06123	SLA-1*11:01:01	90.028	55	−31.7	54 #
SLA08450	SLA-1*11:01:02	90.028	56	−31.7	55 #
SLA08451	SLA-1*11:05	90.305	53	−32.2	56 #
SLA08459	SLA-1*16:03	89.751	58	−32.8	57 #
SLA08445	SLA-1*08:09	88.643	67	−33	58 #
SLA09737	SLA-1*11:11	90.028	57	−33.7	59 #
SLA08443	SLA-1*08:04	89.197	65	−33.8	60 #
SLA08447	SLA-1*08:11	89.474	63	−34.2	61 #
SLA06142	SLA-1*10:02	90.305	51	−34.3	62 #
SLA06125	SLA-1*11:03	89.751	59	−35.4	63 #
SLA08461	SLA-1*19:01	89.197	64	−35.7	64
SLA06143	SLA-1*10:01	89.751	60	−35.8	65 #
SLA08452	SLA-1*11:07	89.474	62	−35.8	66 #
SLA06124	SLA-1*11:02	88.643	68	−36.8	67 #
SLA08453	SLA-1*11:08	88.92	66	−37.5	68 #
SLA08455	SLA-1*11:10	88.366	69	−39.1	69
SLA09717	SLA-1*23:01	88.366	70	−39.1	70

a SLA-1 alleles are sorted based on their sequence similarity and our CAAI method relative to reference SLA-1*04:01:01. All alleles are 361 amino acids in length. SLAs ranked in different position across methods, indicated with #. The sequences of all SLA alleles in these studies are obtained from Immuno Polymorphism Database (https://www.ebi.ac.uk/ipd/licence/). Scores are collected based on our developed algorithm.

One common approach to ranking SLA-1 alleles is simply comparing the entire amino acid sequence to a reference allele, regardless of the position of the non-conserved amino acids or the change in R groups between the substituted amino acids. The percent identity score and rank of the 70 full-length SLA-1 alleles relative to the same reference allele are shown in Table 1. When the rankings were not conserved across both methods, the last column is indicated with #. Approximately 13% of all evaluated SLAs, including SLA-1*04:04, SLA-1*04:05, SLA-1*13:02, SLA-1*06:01, SLA-1*02:02, SLA-1*02:03, SLA-1*19:01, SLA-1*11:10, and SLA-1*23:01 were in the same ranked position in both methods. The majority of SLA-1 alleles had different rankings across both methods; e.g. SLA-1*12:03, SLA-1*12:01, SLA-1*11:09, SLA-1*08:12, SLA-1*11:06, SLA-1*08:18, SLA-1*08:10, SLA-1*10:02, and SLA-1*21:03 differed by eight or more, when ranked across both methods. The ranking of the SLA-2 and SLA-3 alleles based on our CAAI method are shown in Supplementary Table S1. By ranking SLA-1 in this way, we can purposefully select SLA-1 alleles with diverse peptide-binding pocket sequences and better assess epitope diversity for vaccine development.

To confirm that the CAAI method was able to differentiate between similar and diverse alleles, we randomly selected 17 pairs of SLA-1 alleles and showed their ranked score relative to the reference allele based on sequence similarity and the CAAI method (Supplementary Table S2). Docking scores were calculated against a known crystallized epitope using HADDOCK software. The docking scores showed better agreement with the CAAI method (14 out of 17) than with sequence similarity ranking (11 out of 17). Also, we performed pairwise distance analysis as another means to calculate similarity and diversity across the repertoire of functional SLA-1 alleles to the reference allele (Supplementary Table S3).

To further validate the CAAI method, we calculated the Spearman correlation coefficient between our ranking and pairwise distance ranking and the similarity-based ranking. There are different formulas for evaluating correlation coefficient, including the well-known Spearman or Kendall. The correlation coefficient between Table 1, the results of the CAAI ranking and Supplementary Table S3 are calculated and shown in Supplementary Fig. S6a. The calculated Spearman correlation coefficient between our CAAI and the pairwise distance for the same SLA is *r* = 0.9311, and between CAAI and similarity-based is *r* = 0.963 (graph not shown). This shows the positive correlation and further validates the CAAI assessment. The plotted liners regression line of CAAI and Pairwise, as well as between CAAI and similarity-based ranking, are perfectly matched, which indicates a strong correlation. In addition, to address the algorithm’s effectiveness in finding the relation between rank compared to the reference SLAs, we have done blind peptide–protein docking through HDCOK. The 21 random SLA-1 sets were selected, and the crystalized 3QQ3 epitope was docked into their peptide-binding sites. We have compared a group of two SLAs to each other. The results are valid if the epitope is correctly positioned inside the binding site. Notably, docking results depend on many factors, such as the quality of generated models and the accuracy of its search algorithm (Supplementary Fig. S6b). From 21 sets, only four sets were not in agreement when we considered our CAAI ranking. For instance, SLA-1*04:05 and SLA-1*13:02 show −328.8 and −261.5, or SLA-1*04:03 and SLA-1*17:02 show −327.8 and −207.3, which agree with the ranking, but SLA-1*08:18 and SLA-1*08:15 had −230.9 and −272.6, which is not correlated with the ranking (Supplementary Fig. S6).

### 3.2 Using electrostatic potential map within the peptide-binding site to refine selection of diverse SLA-1 alleles

Despite residues being separated by several dozen amino acids in the primary sequence, they may be in close proximity in space and therefore contribute to a localized charge. Thus, two SLA-1 alleles with significant differences in their primary sequences might share comparable electrostatic potential maps throughout the binding groove and may be able to bind similar epitopes. Therefore, apart from the CAAI method, we must consider the charge distribution in the peptide-binding site to identify diverse SLA-1 alleles within a population (Nelson 2005).

To accomplish this, we selected SLA-1 alleles with high, moderate, and low CAAI ranking relative to the SLA-1*04:01:01 reference allele and subjected each to homology modeling to estimate their conformations (Jurrus *et al.* 2018). Next, the SLA-1 alleles were submitted to the APBS electrostatics plugin in PyMol to quantify the electrostatic charge distribution throughout their surfaces (Supplementary Fig. S1). The electrostatic potential maps are illustrated such that negatively charged sites are red, positively charged regions are blue, and areas with a neutral charge are grey (detailed in Supplementary Fig. S3). The electrostatic potential maps for the SLA-1 alleles ranked 1–4 and 66–70 in the CAAI method are depicted in Fig. 3. Despite these rankings, the alleles show very dissimilar electrostatic charges both overall and within the peptide-binding groove (indicated by the green box) (Fig. 3 and Supplementary Fig. S3).

We manually curated the electrostatic maps of alleles with high, moderate, and low CAAI ranking (Table 1) to select SLA-1 alleles with highly negative, highly positive, modestly negative peptide, or moderately positive binding pockets (Supplementary Fig. S2). By using both criteria, we selected eight SLA-1 alleles (SLA-1*06:02, SLA-1*04:04, SLA-1*07:03, SLA-1*04:02, SLA-1*11:10, SLA-1*23:01, SLA-1*08:09, and SLA-1*08:05) to serve as representative “diverse” SLA-1 alleles.

### 3.3 Applying the structure–function relation of SLA-1 peptide-binding pockets to predict T-cell epitopes

None of the eight diverse SLA-I alleles are part of the NetMHCPan4.1 repertoire, nor are they used in ANN 4.0, SMMPMPEC, and SMM (Peters and Sette 2005, Moutaftsi *et al.* 2006, Nielsen *et al.* 2007, Zhang *et al.* 2009, Andreatta and Nielsen 2016). Because we aimed to identify epitopes within the PRRSV M protein that bind strongly to our eight SLA-1 alleles, we needed to perform three separate steps: (i) identify the epitope repertoire in PRRSV M protein, (ii) create a molecular scale that we can use as a reference by generating a database of the docking energy between the SLA-1 alleles within the NetMHCPan4.1 repertoire, and (iii) perform docking analysis on the M protein epitopes and our eight SLA-I alleles to select the strongest binding epitopes.

### 3.4 Identify the epitope repertoire in the PRRSV M protein

The recommended tool by IEDB (https://www.iedb.org/) to select T-cell epitopes is NetMHCpan 4.1, which predicts the potential epitopes within an antigen against 12 SLA-1 alleles in its repertoire (Nielsen *et al.* 2007, Reynisson *et al.* 2020). We used NetMHCpan4.1 to break down the PRRSV M protein into 9-mer epitopes and sorted them according to their binding specificity/strength for the 12 SLA-1 alleles in the NetMHCpan4.1 repertoire (Supplementary Table S4). The 174 amino acids long PRRSV M protein was predicted to have ∼166 9-mer peptides. Results showed that while some epitopes only strongly bind to 1 or 2 of the 12 SLA-1 alleles, some predicted epitopes have strong binding affinity for several alleles. For example, STAPQKVLL is predicted to be a strong epitope through binding to SLA-1*01:01, SLA-1*11:01, and SLA-1*12:01. Likewise, ITYTPVMIY is predicted to bind SLA-1*02:01, SLA-1*02:02, SLA-1*04:01, SLA-1*05:01, SLA-1*07:01, SLA-1*07:02, SLA-1*08:01, and SLA-1*12:01 (Supplementary Table S4).

### 3.5 Generate a molecular scale of the docking energies between SLA-I alleles and strong binding epitopes predicted using NetMHCpan4.1

We subjected the epitope/SLA-1 binding pairs in Step 1 to homology modeling followed by peptide–protein docking through HADDOCK to obtain estimated binding energy data (Table 2). For all docking results, the conformations of the complexes were compared to the crystal structure of the reference SLA-1 allele and its bound epitope. The average binding energy (i.e. docking score) for epitopes binding to the 12 SLA-1 alleles was approximately −77.4, and the data ranged from −43.1 (ITYTPVMIY binding to SLA-1*04:01) to −112.4 (YSAIETWKF binding to SLA-1*13:01). These data established a molecular scale of binding energies expected from epitopes that bind strongly to SLA-1 peptide-binding pockets. The antigenicity of the strong binding epitopes was evaluated by submitting these epitopes to the VaxiJen (Doytchinova and Flower 2007) tool (Table 2).

**Table 2.** Docking of predicted strong binders.^a^

M protein epitopes	SLA-1 strong binder	Binding energy	EpitopeAA	Antigenicity score
AANDNHAFV	*01:01	−95.6	126	0.404; Prob. Ag
ANDNHAFVV	*01:01	−75.7	127	0.553; Prob. Ag
APQKVLLAF	*07:01	−79.5	14	0.247; Prob. Non-Ag
	*07:02	−81.6	14	
AVKQGVVNL	*12:01	−66.9	161	0.572; Prob. Ag
GVYSAIETW	*05:01	−48.6	84	−0.503; Prob. Non-Ag
	*02:02	−86.5	84	
	*1301	−88.8	84	
	*02:01	−80	84	
	*06:01	−77.6	84	
	*08:01	−85.1	84	
IAANDNHAF	*1301	−90.1	125	0.556; Prob. Ag
	*06:01	−90.5	125	
ITYTPVMIY	*05:01	−54.1	24	0.839; Prob. Ag
	*02:02	−96.2	24	
	*07:01	−73.6	24	
	*04:01	−43.1	24	
	*07:02	−81.7	24	
	*02:01	−90.3	24	
	*12:01	−95.3	24	
	*08:01	−92.5	24	
MGAVVALLW	*05:01	−81.7	75	0.548; Prob. Ag
RLLGLLHLL	1*12:01	−82	40	0.332; Prob. Non-Ag
STAPQKVLL	*01:01	−54.8	12	0.0803 Prob. Non-Ag
	*06:01	−49	12	
	*12:01	−68	12	
	*11:01	−55	12	
STNRVALTM	*02:02	−60.7	67	0.509; Prob. Ag
	*04:01	−63.6	67	
	*02:01	−55.1	67	
	*06:01	−54	67	
TLVPGLKSL	*12:01	−62.9	146	0.870; Prob. Ag
VLLAFSITY	*02:02	−79.4	18	1.292; Prob. Ag
	*07:01	−71.3	18	
	*07:02	−78	18	
	*02:01	−79.2	18	
	*08:01	−85.8	18	
VPGLKSLVL	*11:01	−57.2	148	0.463; Prob. Ag
YSAIETWKF	*02:02	−110.2	86	−0.259; Prob. Non-Ag
	*13:01	−112.4	86	
	*02:01	−108.5	86	
	*06:01	−109	86	
			

a The binding energy of strong epitopes docked into the 12 SLA-1 alleles predicted by NetMHCpan 4.1. All epitopes were subjected to homology modeling followed by peptide–protein docking through HADDOCK. The antigenicity has been evaluated by Vaxijen. M protein epitopes, shows the sequence of the predicted epitopes. SLA-1 strong binder, shows the SLA information. Binding Energy, HADDOCK score. Epitope AA position, the position of the first residue of the epitope. Antigenicity score, obtained from Vaxijen with a threshold of 0.4.

We selected the M protein epitopes predicted to bind weakly to all or some 12 SLA-1 alleles in the NetMHCpan4.1 repertoire and used them to identify epitopes that bind strongly to the eight diverse SLA-1 alleles. Then, performed homology modeling followed by peptide–protein docking through HADDOCK to obtain the estimated binding energy data of M protein epitopes with our eight SLA-1 alleles (Table 3). The average binding energy for M protein epitopes binding to the eight diverse SLA-1 alleles was approximately −77.3, and the data ranged from −52.2 (STNRVALTM binding to SLA-1*08:05) to −121.6 (FGYMTFVHF binding to SLA-1*06:02), which showed comparable binding energy to the docking score of NetMHCpan4.1strong binders. These epitopes were also subjected to VaxiJen to evaluate their antigenicity (Table 3).

**Table 3.** Prediction of strong epitopes based on evaluations of docking results.^a^

M protein epitopes	Strongly bound SLA I	Binding energy	Epitope AA	Antigenicity score
AANDNHAFV	*08:05	−62.5	126	0.432; Prob. Ag
AFTFGYMTF	*04:04	−85.9	54	1.380; Prob. Ag
AFVVRRP	*04:04	−77.4	132	1.380; Prob. Ag
AFVVRRPGS	*08:09	**−86.2**	132	−0.303; Prob. Non-Ag
FGYMTFVHF	*06:02	**−121.6**	57	1.520; Prob. Ag
FHPIAANDN	*23:01	−92.8	122	0.833; Prob. Ag
FSITYTPVM	*04:02	−82.3	22	1.406; Prob. Ag
HHVESAAGF	*08:05	−96	114	0.574; Prob. Ag
IAANDNH	*08:09	−78	125	0.844; Prob. Ag
IAANDNHAF	*04:02	−82.6	125	0.557; Prob. Ag
	*04:02	−82.6	125	
IFLNCAFTF	*04:04	−86.2	49	0.369; Prob. Non-Ag
KYILAPAHH	*04:02	−71.9	107	0.093; Prob. Non-Ag
LGLLHLLIF	*04:02	−87.4	42	0.645; Prob. Ag
LNCAFTFGY	*11:10	−88.5	51	1.000; Prob. Ag
PIAANDNH	*11:10	−66.8	124	0.704; Prob. Ag
QGVVNLVKY	*23:01	−78	164	−0.040; Prob. Non-Ag
RLCLLGRKY	*06:02	−79.3	100	1.854; Prob. Ag
SAIETWKFI	*04:04	−72.2	87	−0.401; Prob. Non-Ag
SITYTPVMI	*08:09	−56.9	23	1.295; Prob. Ag
STAPQKVLL	*04:04	−68.2	12	0.079; Prob. Non-Ag
	*11:10	−65.7	12	
STNRVALTM	*08:09	−71.8	67	0.509; Prob. Ag
	*08:05	−52.2	67	
TYTPVMIY	*04:04	−76.2	25	0.245; Prob. Non-Ag
	*23:01	−89.9	25	
	*11:01:01	−80.7	25	
TYTPVMIYA	*04:02	−54.6	25	0.161; Prob. Non-Ag
	*11:10	−76.7	25	
	*23:01	−77.5	25	
	*08:09	−63.4	25	
	*08:05	−63	25	
	*11:01:01	−65.7	25	
VHFESTNRV	*04:04	−67.5	63	1.374; Prob. Ag
VLLAFSITY	*11:10	−93.5	18	1.292; Prob. Ag
VSRGRLLGL	*08:05	−53.4	36	0.953; Prob. Ag
VVALLWGVY	*08:05	−67.4	78	0.672; Prob. Ag
WGVYSAIET	*23:01	−94	83	0.520; Prob. Ag
YILAPAHHV	*23:01	−99.6	108	0.142; Prob. Non-Ag
YSAIETWKF	*06:02	−86.4	86	
	*04:04	−100.5	49	
	*04:02	−97.2	86	−0.259; Prob. Non-Ag
YTPVMIYAL	*04:02	−78.5	26	0.210; Prob. Non-Ag
VKQGVVNLV	*07:03	−69.4	162	0.463; Prob. Ag
KAVKQGVVN	*07:03	−60.2	160	0.471; Prob. Ag
PGLKSLVLG	*07:03	−55.5	149	1.295; Prob. Ag
SAAGFHPIA	*07:03	−71.8	118	0.859; Prob. Ag

a Weak binders to 12 NetMHCpan 4.1 SLA-1 alleles were docked against the 8 SLA-1 diverse alleles. All epitopes were subjected to homology modeling followed peptide–protein docking through HADDOCK. The antigenicity has been evaluated by submitting these epitopes to Vaxijen tool. For each SLA-1 allele, two strong binders were selected based on their docking score in green.

**Figure 4.**
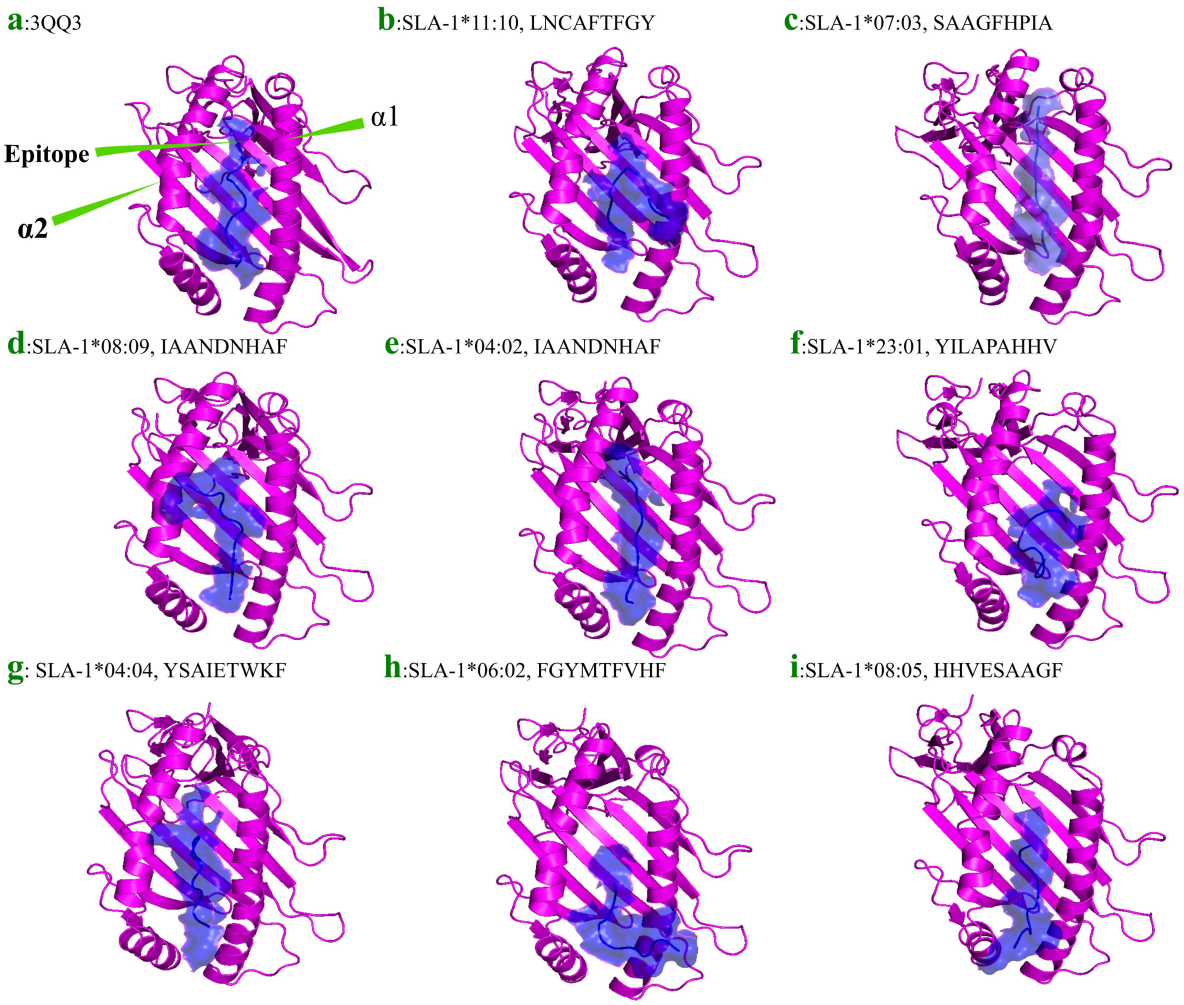
Comparison of docking poses to the crystalized epitope. Eight SLA-1 alleles with their docked epitopes are compared to crystalized SLA-1*04:01-PDB: 3qq3 reference allele and its crystallized epitope. The binding energies are shown in Table 3, and protein–peptide affinities are shown in Table 4. The α1 and α2 helices are shown for the first SLA (a: 3qq3); all other SLAs (b-i) are superimposed to the first one to replicate the same position of their α1 and α2 helices. The quality of dockings shows complexes are comparable to the crystalized one. In all cases, the SLA-1 is colored in magenta, and the surface representation of the epitope is shown in blue. (a) SLA-1*04:01, (b) SLA-1*11:10 (LNCAFTFGY), (c) SLA-1*07:03 (SAAGFHPIA), (d) SLA-1*08:09 (IAANDNHAF), (e) SLA-1*04:02 (IAANDNHAF), (f) SLA-1*23:01 (YILAPAHHV), (g) SLA-1*04:04 (SAIETWKF), (h) SLA-1*06:02 (FGYMTFVHF), and (i) SLA-1*08:05 (HHVESAAGF), respectively.

We aimed to narrow down the two best epitopes predicted to bind to the eight diverse SLA-1 alleles. HADDOCK generates up to eight clusters for each epitope-SLA complex composed of at least four docked epitope-SLA poses. After selecting two strong binders according to HADDOCK scores, two clusters containing complexes with the strong scores were subjected to protein–protein affinity evaluation based on the PRODIGY algorithm (Table 4). The sums for each cluster were considered for the final selection such that epitopes FGYMTFVHF (−80.5 kcal/mol), YSAIETWKF (−81.7 kcal/mol), IAANDNHAF (−82.5 kcal/mol), LNCAFTFGY (−85.7 kcal/mol), YILAPAHHV (−81.8 kcal/mol), IAANDNHAF (−82.7 kcal/mol), HHVESAAGF (−77 kcal/mol), and SAAGFHPIA (−82.8 kcal/mol) showed high affinity toward SLA-1*06:02, SLA-1*04:04, SLA-1*04:02, SLA-1*11:10, SLA-1*23:01, SLA-1*08:09, SLA-1*08:05, and SLA-1*07:03, respectively (Table 4). Finally, we show the quality of the docking of the top binding epitope with each of the eight SLA-1 alleles (Fig. 4b–i) relative to the reference allele and its crystalized epitope (Fig. 4a).

**Table 4.** Binding affinity of epitopes predicted to bind to the eight representative SLA-1 alleles.^a^

SLA-1	Sequence	1–1	1–2	1–3	1–4	2–1	2–2	2–3	2–4	Sum
*04:02	YSAIETWKF	−9.7	−9.3	−9.4	−9.7	−11.3	−9.3	−11	−11.8	−81.3
*04:02	**IAANDNHAF**	−10.8	−10.3	−9.6	−10.2	−10.8	−11.5	−9.4	−9.9	−82.5
*04:04	IFLNCAFTF	−9.9	−10.2	−10.1	−9.5	−9.9	−10.6	−10	−9.9	−80.2
*04:04	**YSAIETWKF**	−10.9	−11.3	−10.4	−10.4	−9.4	−9.4	−10	−9.8	−81.7
*06:02	YSAIETWKF	−10.1	−9.3	−9.8	−9.7	−9.9	−9.3	−10	−8.7	−76.9
*06:02	**FGYMTFVHF**	−10.2	−9.8	−10.4	−10.9	−10.3	−9.7	−9.9	−9.3	−80.5
*07:03	KAVKQGVVN	−10.4	−10.5	−9.2	−10	−9.4	−9.6	−9.3	−10.1	−78.5
*07:03	**SAAGFHPIA**	−10.8	−9.7	−10.9	−10.5	−10.4	−10.3	−9.9	−10.3	−82.8
*08:05	VVALLWGVY	−9.6	−9.5	−9.9	−10.2	−8	−8.4	−8.2	−9	−72.8
*08:05	**HHVESAAGF**	−9.3	−9.5	−9.3	−9.3	−10.7	−10.3	−9.8	−8.8	−77
*08:09	AFVVRRPGS	−10	−9.8	−9.4	−9.3	−10.2	−11.2	−11	−10.7	−81.5
*08:09	**IAANDNHAF**	−11.3	−8.8	−10.6	−10.9	−10.5	−10.2	−9.7	−10.7	−82.7
*11:10	VLLAFSITY	−11.3	−10.4	−10.2	−10.1	−9.5	−9.6	−8.7	−9.6	−79.4
*11:10	**LNCAFTFGY**	−10.7	−10.3	−12	−10.9	−10.6	−11.2	−9.8	−10.2	−85.7
*23:01	WGVYSAIET	−10.3	−10.6	−10.2	−11.4	−8.8	−9.3	−10	−9.2	−80
*23:01	**YILAPAHHV**	−11	−10	−10.5	−10.4	−9.5	−10.1	−11	−9.5	−81.8

a Sixteen selected epitopes that bind to the eight SLA-1 alleles were subjected to PRODIGY binding affinity assessment, and the eight epitopes with the strongest binding affinity (kcal/mol) are shown in bold font.

Because the accuracy of docking is highly dependent on the 3D structures, we mapped Ramachandran plots for the epitopes and SLA-1 conformations (Supplementary Figs S4 and S5). The Ramachandran plots for the epitopes ranged from 71% to 100% favorable-allowed, and the Ramachandran plots for the eight diverse SLA-1s were >92% (data not shown). The homology modeled conformations of the eight SLA-1 alleles are also shown in Supplementary Fig. S5. These results give us confidence that the 3D structures generated by homology modeling are likely accurate.

## 4 Discussion

Due to the enormous diversity in MHCs, selecting functional alleles that can be used to develop highly effective vaccines cannot be easily achieved in most species. Many MHC/SLAs share more than 90% similarity, but even with this high degree of overall homology, the peptide-binding site can differ by up to six amino acids. Changes in epitope or SLA allele amino acid sequences have the potential to significantly affect (or conversely to have a minimal impact) the binding of the epitope to the SLA or the interaction of the SLA with the T-cell receptor. Ultimately, how structural investigation benefits epitope prediction and the best way to achieve the prediction needs to be better elucidated. Because most prediction tools are limited in the repertoire of MHCs/SLAs available to them, this restricts effective vaccine development. While artificial intelligence can be employed to predict epitope-MHC binding interactions (Wang and Pai 2014, Reynisson *et al.* 2020), computational-structural consideration (i.e. MDS and docking simulation) can greatly improve finding epitopes that can bind strongly to a wide range of MHC/SLA alleles.

In this study, we investigated a new approach to assess SLA-1 similarity by assigning weighting criteria to non-conserved amino acids based on their position and differences in the R groups’ characteristics (charge, size, polarity, etc.). The MDS result determined that most SLA-1 residues contributing to epitope interactions are located at the α1 and α2 regions and they are either charged or aromatic residues. A fast and efficient algorithm was developed to accurately calculate the scores for full-length SLA-1 alleles. The electrostatic potential maps showed that even when some of the SLA-1 alleles share a close similarity ranking, the charge in the peptide-binding groove can be diverse, so we manually curated a list of eight SLA-1 alleles to represent those with diverse sequences and negative, positive, or relatively neutral charges in the peptide-binding groove. These eight SLA-1 alleles were then used to represent SLA availability within an outbred population.

We performed homology modeling, protein–peptide docking, and protein–peptide affinity evaluations to identify epitopes predicted to bind strongly to eight SLA-1 alleles. The discovered epitopes include FGYMTFVHF, YSAIETWKF, IAANDNHAF, LNCAFTFGY, YILAPAHHV, IAANDNHAF, HHVESAAGF, and SAAGFHPIA by showing high binding affinity toward SLA-1*06:02, SLA-1*04:04, SLA-1*04:02, SLA-1*11:10, SLA-1*23:01, SLA-1*08:09, SLA-1*08:05, and SLA-1*07:03, respectively. Some of these epitopes were predicted to bind more strongly to our selected pools of SLA-1 alleles relative to the epitopes predicted by NetMHCpan 4.1 to bind strongly to its 12 SLA-1 alleles repertoire. We are aware that NetMHCpan provides score in addition to %Rank, we only considered the %Rank as it is advised.

Our approach was used to rank SLAs and find epitopes that bind strongly to a unique set of SLA-1 alleles. This approach can be employed in choosing amongst dozens to several hundred available SLAs to complement other tools or an independent approach to identify epitopes that bind strongly to alleles not part of the repertoire in available epitope prediction tools. Wet lab experiments and eluted ligand assays are now needed to establish whether this approach leads to superior vaccine development for outbred animals and further improves the SLA ranking system that might be useful for other MHCs.

## Supplementary Material

btad590_Supplementary_Data

## Data Availability

The data underlying this article are available in the online Supplementary Material.
