# Supplementary material for: Using a novel structure/function approach to select diverse swine major histocompatibility complex 1 alleles to predict epitopes for vaccine development
Source: Bioinformatics. 2023 Sep 22;39(10):btad590. doi: 10.1093/bioinformatics/btad590 (PMC10551226; doi:10.1093/bioinformatics/btad590)
Supplement: btad590_Supplementary_Data [file btad590_supplementary_data.pdf]

Supplementary Material

```
clc
clear

for n=1:361
  dx{n,1}=[];
  dx{n,2}=[];
  dx{n,3}=[];
  dx{n,5}=[];
end

xlswrite('Book1.xlsx',dx)

 %a=[1 4 5;8 7 8 ]
%[sa,so]=find(a==4)
fid=fopen('system.txt');
A=textscan(fid, '%s', 'delimiter', '\n');
%C1=textscan(fid, '%s', 'delimiter', ' ');
di=numel(A{1,1});
px{2,5}=0;
px{3,5}=0;
px{4,5}=0;
px{5,5}=0;
px{6,5}=0;
for j=1:2
   x{1,j}=A{1}{j}  ;
```

```
end
 s=1;
 p1=1;
 p2=2;
 Namino=0;
 POS=[-0.1 -0.25 -0.5 -0.75 -0.75
      -0.25 -0.1 -0.5 -0.75 -0.75
      -0.5 -0.5 -0.1 -0.75 -0.75
      -0.75 -0.75 -0.75 -0.1 -1
      -0.75 -0.75 -0.75 -1 -0.1
      ];
px{1,1}=[];
for k=1:1

   x1=x{1,p1};
   x2=x{1,p2};
   p1=p2+1;
   p2=p2+2;

   L1=length(x1);
   L2=length(x2);


   for i=1:L1
      res=strcmp(x1(i), x2(i));
      g1=0;
      g2=0;
      if res==0

         % payda kardan radif position
```

```
        %g1

        if x1(i)=="G" || x1(i)=="A" || x1(i)=="V" ||
x1(i)=="L" || x1(i)=="M" || x1(i)=="I"

            g1=1;

        else if x1(i)=="S" || x1(i)=="T" || x1(i)=="C" ||
x1(i)=="P" || x1(i)=="N" || x1(i)=="Q"
             g1=2;

        else if x1(i)=="F" || x1(i)=="Y" || x1(i)=="W"
             g1=3;

        else if x1(i)=="K" || x1(i)=="R" || x1(i)=="H"
             g1=4;

        else if x1(i)=="D" || x1(i)=="E"
             g1=5;

            else
                g1=0;
            end
            end
            end
            end
         end
         %g2
            if x2(i)=="G" || x2(i)=="A" || x2(i)=="V" ||
x2(i)=="L" || x2(i)=="M" || x2(i)=="I"
```

```
            g2=1;

        else if x2(i)=="S" || x2(i)=="T" || x2(i)=="C" ||
x2(i)=="P" || x2(i)=="N" || x2(i)=="Q"
             g2=2;

        else if x2(i)=="F" || x2(i)=="Y" || x2(i)=="W"
             g2=3;

        else if x2(i)=="K" || x2(i)=="R" || x2(i)=="H"
             g2=4;

        else if x2(i)=="D" || x2(i)=="E"
             g2=5;

            else
                g2=0;
            end
            end
            end
            end
            end
            if g1~=0 && g2~=0
              sum1=POS(g1,g2);
              px{s,3}=sum1;
              Namino=Namino+1;
            end

         px{s,1}=[x1(i), x2(i)];
```

```
        px{s,2}=i;
        s=s+1;


    end

switch(i)
    case 22
            score=-0.1*Namino;
            Namino=0;
            px{2,5}=score;
    case 203
            score=-0.5*Namino;
            Namino=0;
            px{3,5}=score;
    case 304
            score=-0.1*Namino;
            Namino=0;
            px{4,5}=score;
    case 329
            score=-0.2*Namino;
            Namino=0;
            px{5,5}=score;
   case 361
            score=-0.1*Namino;
            Namino=0;
            px{6,5}=score;
end
end
```

```
  if i>0 && i<22
       score=-0.1*Namino;
           px{2,5}=score;
  else if i>22 && i<203
          score=-0.5*Namino;
           px{3,5}=score;

      else if i>203 && i<304
           score=-0.1*Namino
           px{4,5}=score;
          else if i>304 && i<329
               score=-0.2*Namino;
               px{5,5}=score;
              else if i>329 && i<361
                   score=-0.1*Namino;
                   px{6,5}=score;
                  end
              end
          end
      end

  end
  sumScore=px{2,5}+px{3,5}+px{4,5}+px{5,5}+px{6,5}
  px{7,5}=sumScore;
 %sumGroup=sum(px{5,1})
B2 = cellfun(@sum,px(:,3));
sumGroup=sum(B2)
 px{9,5}=sumGroup;
xlswrite('Book1.xlsx',px)
```

```
end
```

MATLAB Code that classifies SLA alleles based on the designed scoring functions in Figure 1e.
We need fast and accurate algorithms to effectively process a large amount of data in a limited time to obtain information from massive amounts of data. This algorithm has been developed to consider one reference allele (defined by the user that can be any SLA, or with further improvement/modification, any MHCs) and then compare all other SLAs to that one. First, it sorts the data consisting of SLA proteins and generates an algorithm input. The search-based algorithms for data comparison, as well as sequence similarity scores, are based on FCA algorithm matrices and are recorded, ranked and calculated by our concatenation approach. This algorithm is implemented by MATLAB.

SLA Allele Accession #[a]	SLA Allele Name	CAAI ranking	Electrostatic Charge Modeling[b]
SLA06106	SLA-1*04:01:01	Reference	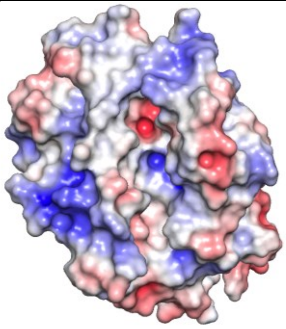
SLA06108	SLA-1*04:04	1	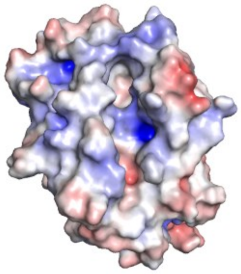
SLA06109	SLA-1*04:05	2	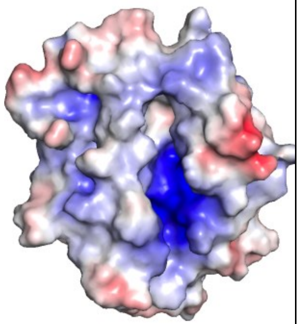

SLA06132	SLA-1*13:02	3	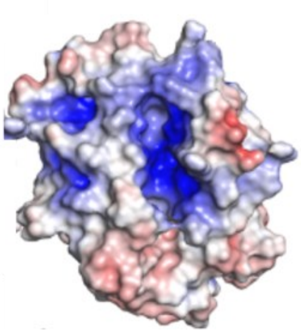
SLA06107	SLA-1*04:03	4	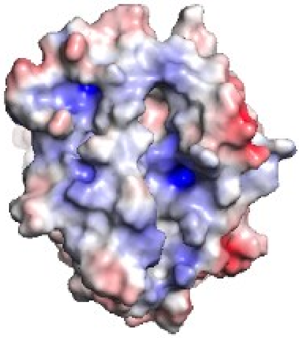
SLA06131	SLA-1*13:01	5	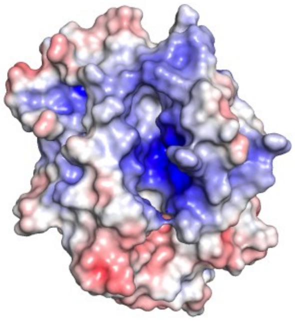
SLA08460	SLA-1*17:02	6	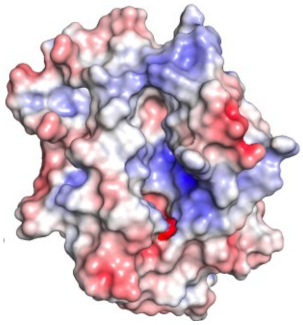

SLA06112	SLA-1*06:02	7	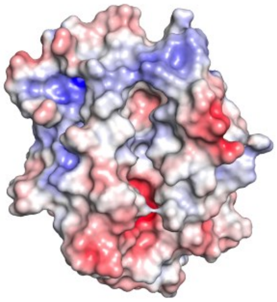
SLA08440	SLA-1*04:02	8	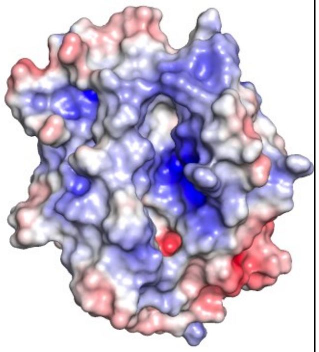
SLA06111	SLA-1*06:01	9	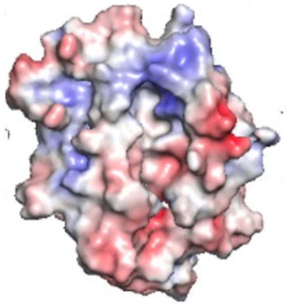
SLA06103	SLA-1*02:01	10	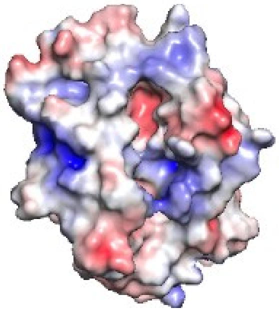

SLA06135	SLA-1*17:01	11	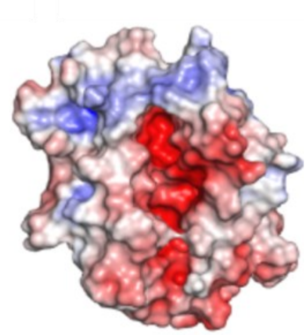
SLA6104	SLA-1*02:02	13	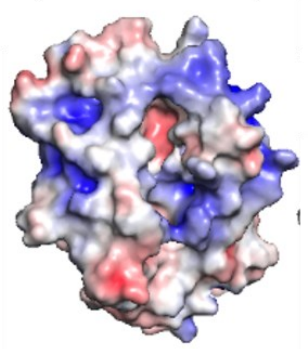
SLA6105	SLA-1*02:03	15	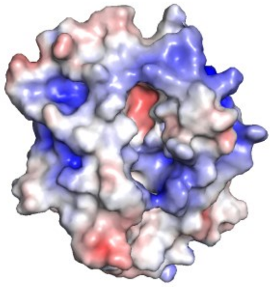
SLA06138	SLA-1*07:03	16	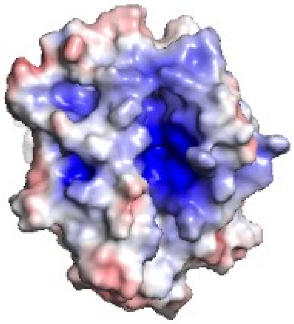

SLA06100	SLA-1*01:01	17	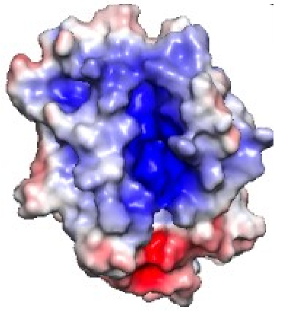
SLA06114	SLA-1*07:02	18	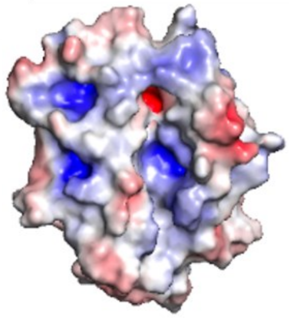
SLA06113	SLA-1*07:01	19	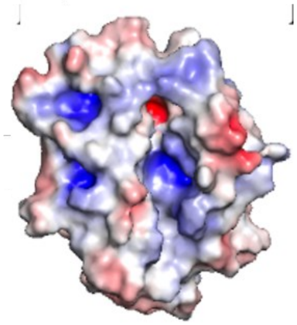
SLA06115	SLA-1*08:01	20	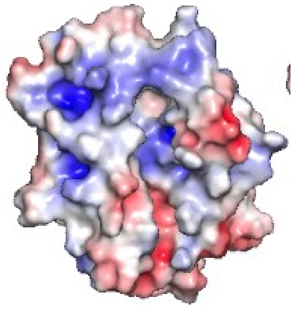

SLA06110	SLA-1*05:01	24	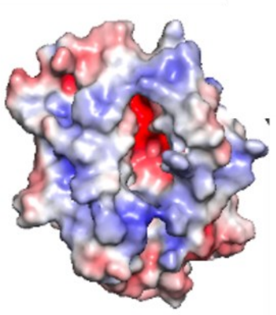
SLA06129	SLA-1*12:03	29	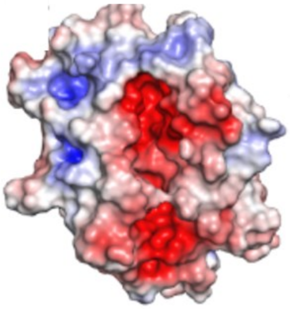
SLA06128	SLA-1*12:01	30	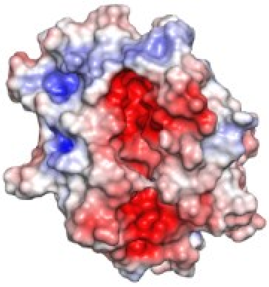
SLA06119	SLA-1*08:07	31	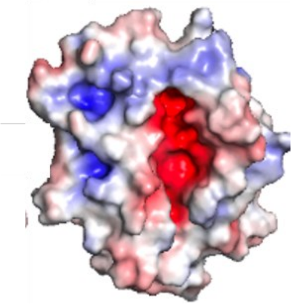
SLA08446	SLA-1*08:10	35	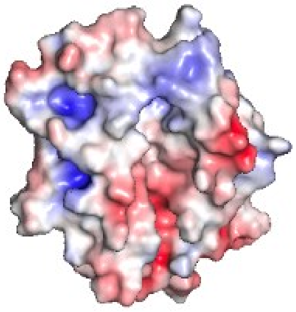

SLA09741	SLA-1*08:18	36	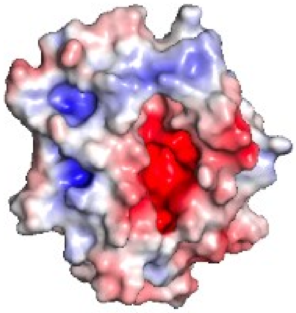
SLA06139	SLA-1*18:01	37	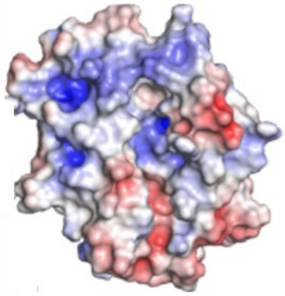
SLA09716	SLA-1*21:03	38	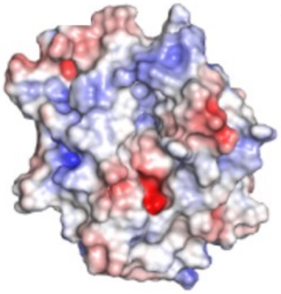
SLA09714	SLA-1*08:15	40	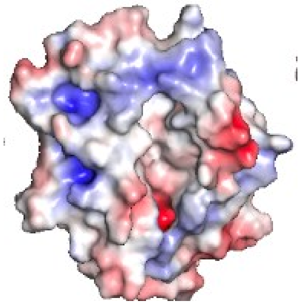
SLA06118	SLA-1*08:13	41	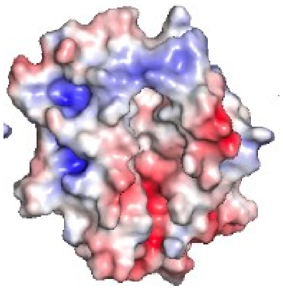

SLA08442	SLA-1*07:06	43	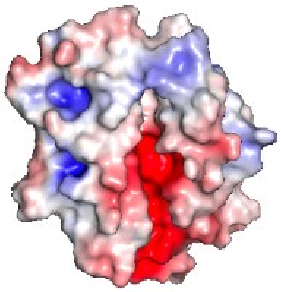
SLA09742	SLA-1*14:04	44	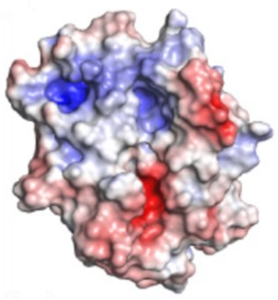
SLA06121	SLA-1*08:08	45	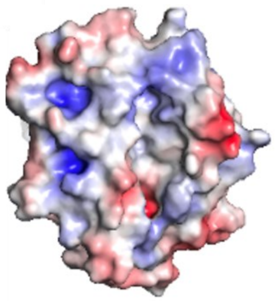
SLA06120	SLA-1*08:03	46	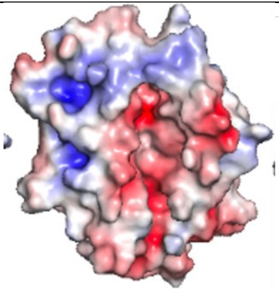
SLA06122	SLA-1*08:05	50	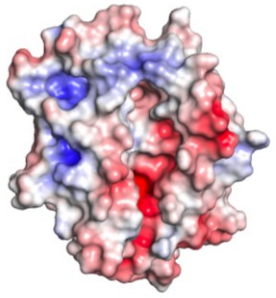

SLA08462	SLA-1*20:01	51	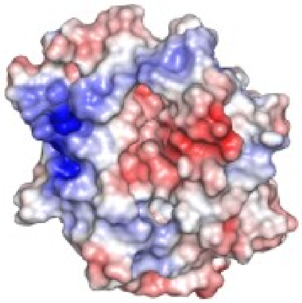
SLA09713	SLA-1*21:01	53	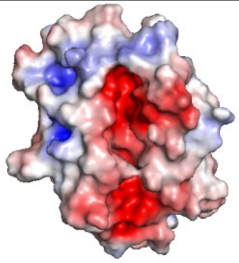
SLA06123	SLA-1*11:01:01	54	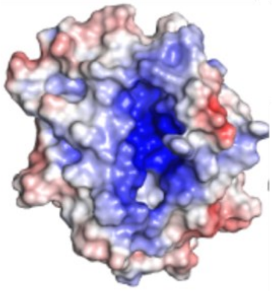
SLA08450	SLA-1*11:01:02	55	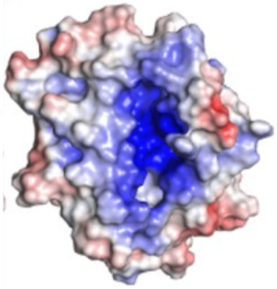
SLA08451	SLA-1*11:05	56	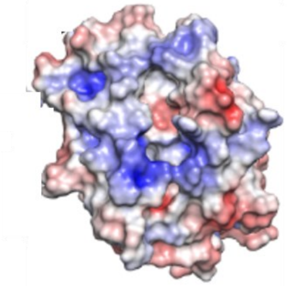

SLA08459	SLA-1*16:03	57	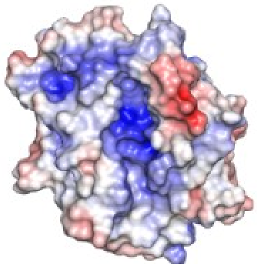
SLA08445	SLA-1*08:09	58	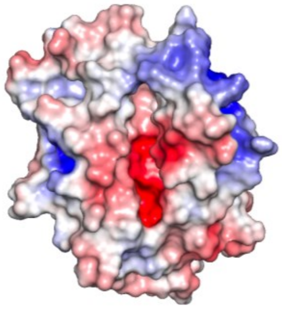
SLA09737	SLA-1*11:11	59	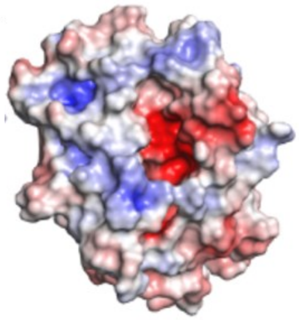
SLA08443	SLA-1*08:04	60	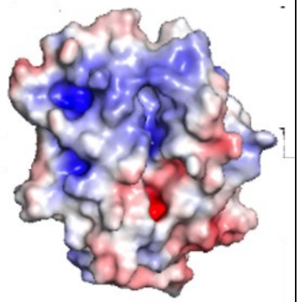
SLA08447	SLA-1*08:11	61	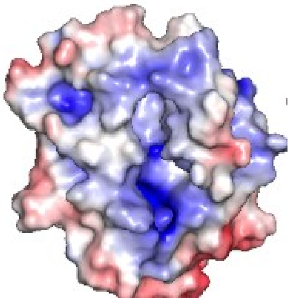

SLA06142	SLA-1*10:02	62	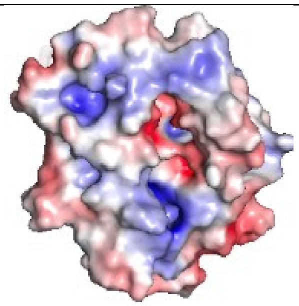
SLA06125	SLA-1*11:03	63	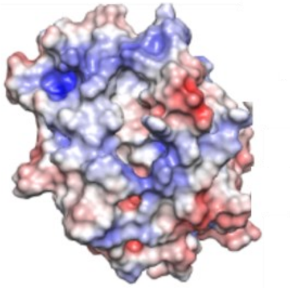
SLA08461	SLA-1*19:01	64	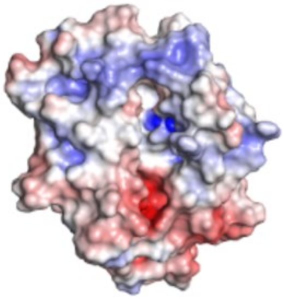
SLA06143	SLA-1*10:01	65	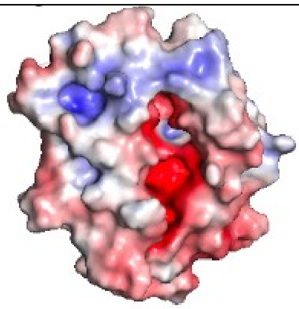
SLA08452	SLA-1*11:07	66	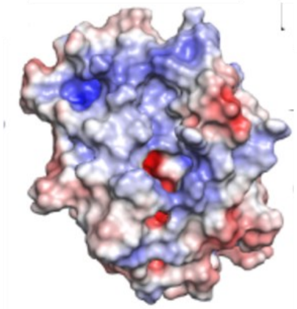

SLA06124	SLA-1*11:02	67	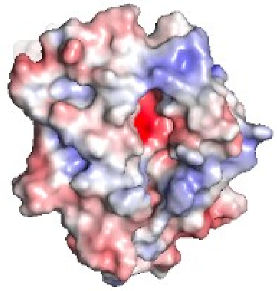
SLA08453	SLA-1*11:08	68	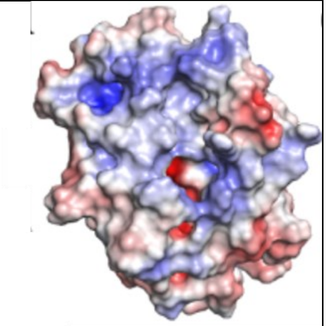
SLA08455	SLA-1*11:10	69	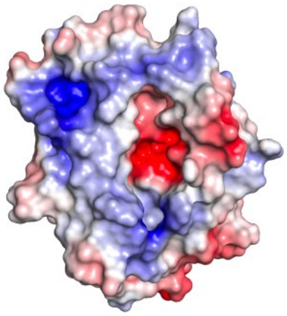
SLA09717	SLA-1*23:01	70	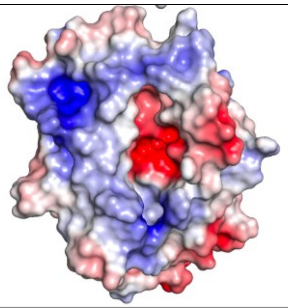

Supplementary Figure 1: **Electrostatic contact maps of SLA-1 alleles**.
[a]Allele accession numbers and names were identified using Immunopolymorphism Database (https://www.ebi.ac.uk/ipd/mhc/group/SLA/). [b]For generating electrostatic maps, the PDB (Protein Data Bank) format was changed to PQR (primary input format for biomolecular structure in APBS package) by submitting the PDB coordinates to the APBS-PDB2PQR software suite. Next, the SLA-1 alleles were submitted to the APBS electrostatics (Adaptive Poisson-Boltzmann Solver) plugin in PyMol to quantify the electrostatic charge throughout the protein. The charge distribution of the binding site (in general, the whole SLA) can be strongly

positive or negative, which displays blue and red colours, respectively, in this evaluation. Deep blue or red are indications of strong positive or negative charges (probably existing of charged or polar residues), and reduced coloration indicates that the neutralized charged region is dominant due to mostly non-polar aliphatic residues. The comparison of the colour and quantification can be seen in **Supplementary Figure 3** for SLA-1 02:01.

Allele Name [a]	CAAI ranking	Electrostatic Charge Modeling[b]	Allele Name [a]	CAAI ranking	Electrostatic Charge Modeling[b]
SLA-1*06:02	7	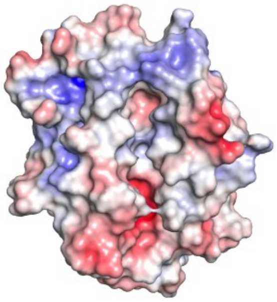	SLA-1*08:05	50	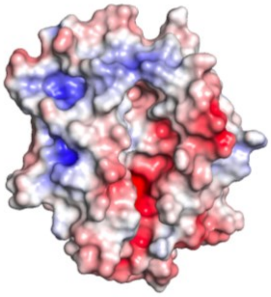
SLA-1*04:02	8	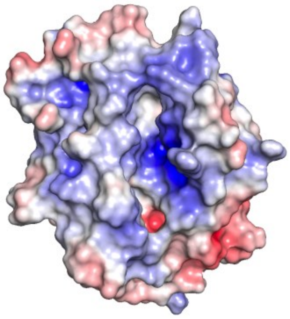	SLA-1*08:09	58	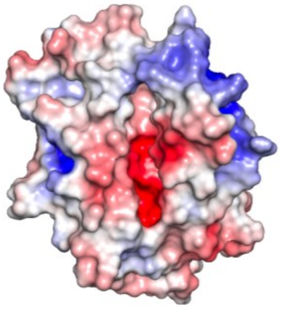
SLA-1*07:03	16	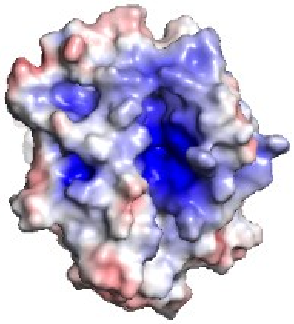	SLA-1*11:10	69	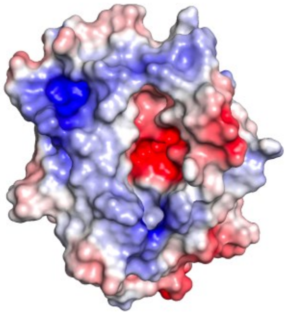

SLA-1*04:04	44	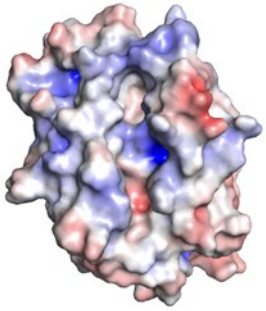	SLA-1*23:01	70	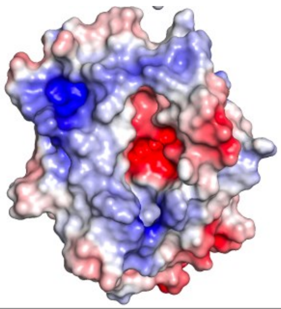

Supplementary Figure 2: CAAI ranking and electrostatic charge distribution of the eight representative diverse SLA-1 alleles. [a]Allele names were identified using Immunopolymorphism Database (https://www.ebi.ac.uk/ipd/mhc/group/SLA/). [b]For generating electrostatic maps, the PDB format was changed to PQR by APBS-PDB2PQR software suite. Next, the SLA-1 alleles were submitted to the APBS electrostatics (Adaptive Poisson-Boltzmann Solver) plugin in PyMol to quantify the electrostatic charged throughout the protein. We selected alleles based on CAAI ranking (close to, moderately close to or far in ranked relative the reference allele). Next, we manually curated these alleles based on charge in the peptide binding groove such that some alleles had highly positive, highly negative or had a weakly positive or negative charge.

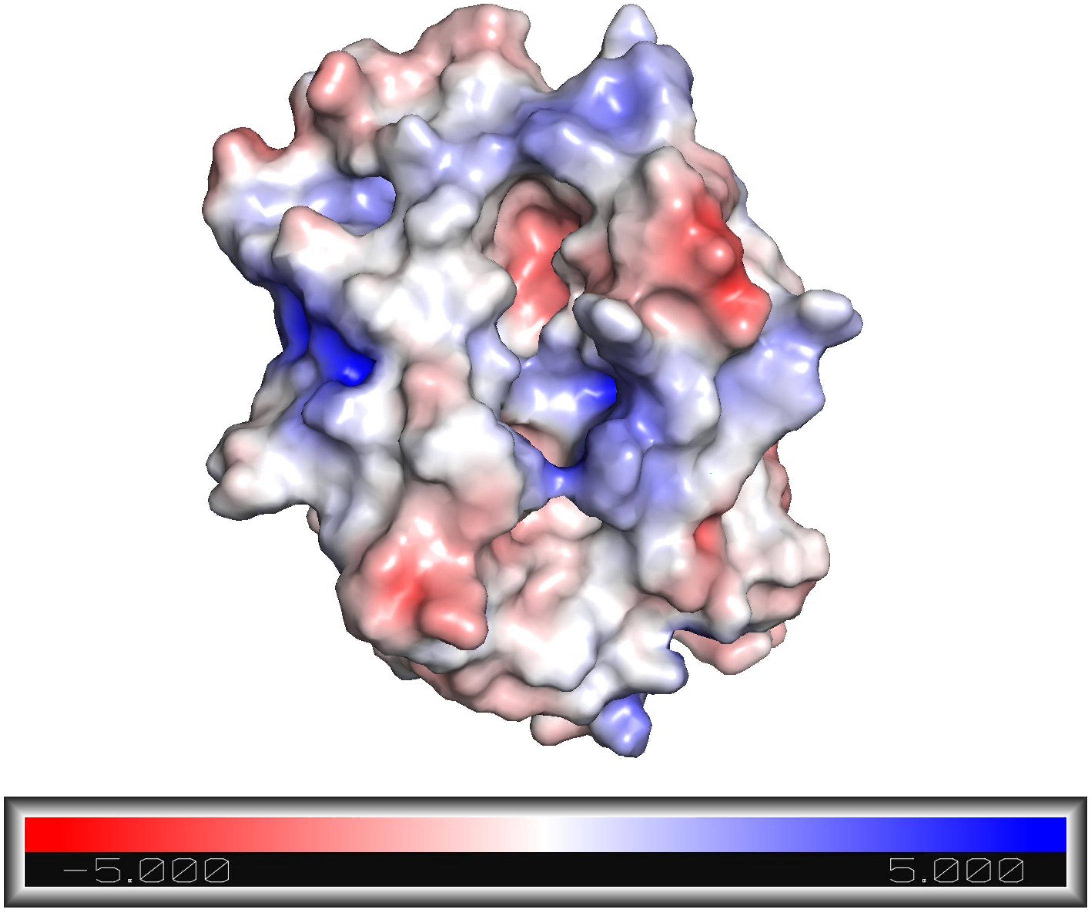

Supplementary Figure 3: The color scale used for **e**lectrostatic charge maps for SLA-1 02:01 as an example and representation applied for all charge distribution map evaluations. The charge distribution of the binding site (in general, the whole SLA) can be strongly positive or negative, which displays blue and red colors, respectively, in this evaluation. Deep blue or red are indications of stronger positive or negative charges (probably existing of charged or polar residues), and losing the color shows the neutralized region is dominant due to mostly non-polar aliphatic residues.

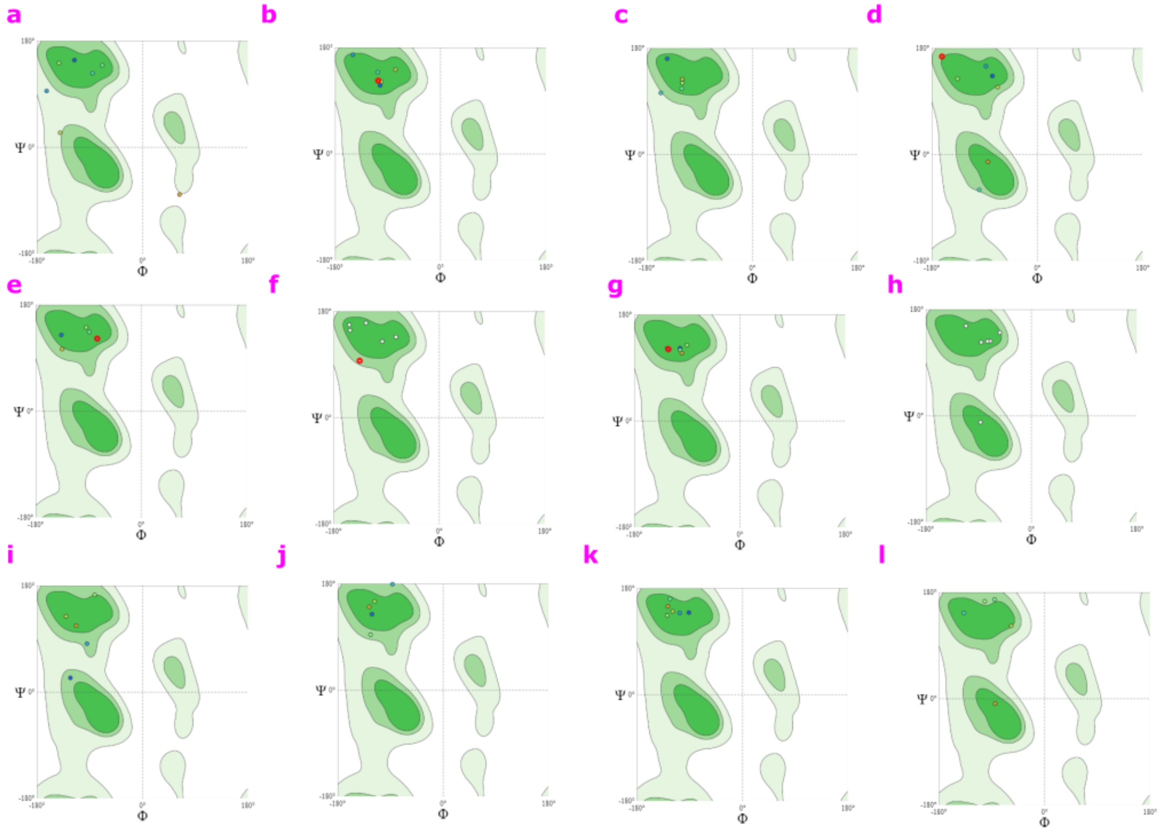

Supplementary Figure 4: Ramachandran plots for generated epitopes. The Ramachandran plots for the epitopes ranged from 71%-100% favorable-allowed. The epitopes are (**a-l**) are GVYSAIETW (71%), ITYTPVMIY (100%), AFVVRRP (80%), ANDNHAFVV (85%), IAANDNH (100%), PIAANDNH (100%), RLLGLLHLL (100%), STAPQKVLL (85%), TLVPGLKSL (71%), TYTPVMIY (83%), TYTPVMIYA (100%) and VPGLKSLVL (85%).

[a]Allele names	Ramachandran favored %	Homology modeled conformation (SWISS-MODEL)
SLA-1*0404	98.1	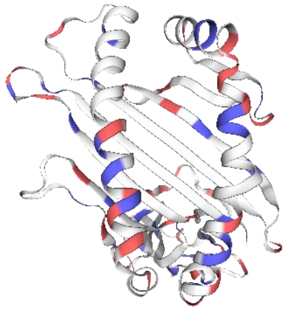
SLA-1*0404	97.4	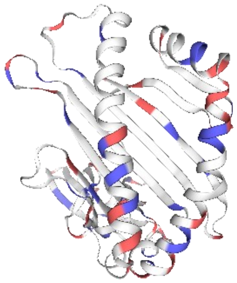
SLA-1*1302	97	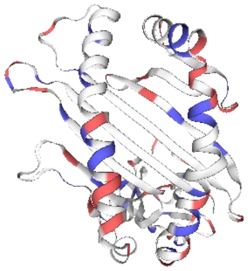

SLA*1-403	96	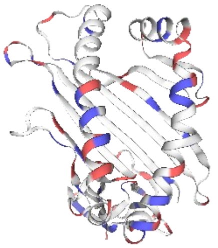
SLA*1-0805	94.2	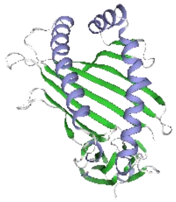
SLA*1-0602	97.8	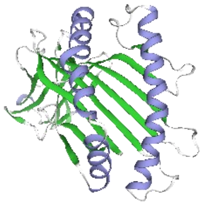
SLA*1-0402	97.2	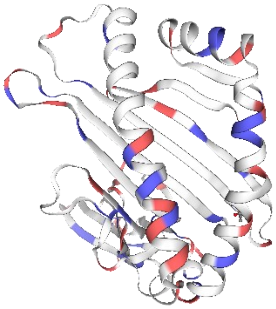

SLA*1-0601	97.5	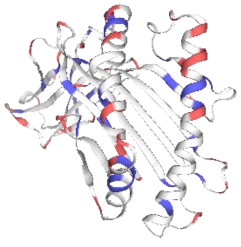
SLA*1-0809	97.5	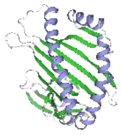
SLA*1-1110	98.1	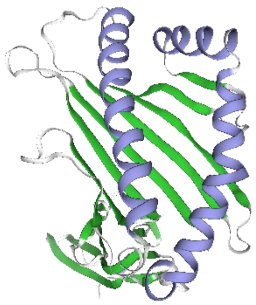
SLA*1-2301	98.2	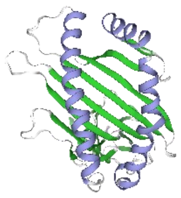

SLA*1-0703	98.15	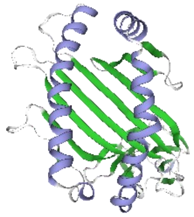
SLA*1-0101	98	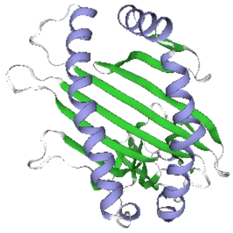
SLA*1-0801	97.4	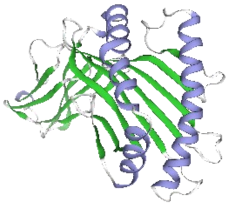
SLA*1-0803	97.5	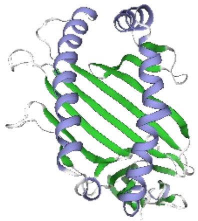

Supplementary Figure 5: The Ramachandran scores and conformations of generated SLAs in this study. [a]Allele names were identified using Immunopolymorphism Database (https://www.ebi.ac.uk/ipd/mhc/group/SLA/). The 3D structure of SLA-1 alleles and selected

epitopes were modeled and evaluated through SWISS-MODEL (https://swissmodel.expasy.org/). Differences in coloration are not relevant.

SLA-2[a]	CAAI Score relative to SLA-2*04:01	SLA-3[a]	CAAI Score relative to SLA-3*04:01
SLA-2*05:03|364	-33	SLA-3*03:07|361	-23.3
SLA-2*09:02|364	-31.8	SLA-3*03:03|361	-17.4
SLA-2*04:09|364	-10	SLA-3*05:04|361	-18.2
SLA-2*10:03|364	-34.6	SLA-3*08:01|361	-39.05
SLA-2*10:06|364	-32.8	SLA-3*05:02|361	-20.5
SLA-2*05:08|364	-29.7	SLA-3*05:07|361	-15.95
SLA-2*13:02|364	-29	SLA-3*05:03:02|361	-18.15
SLA-2*11:01:01|364	-25	SLA-3*05:05|361	-19.75
SLA-2*04:05|364	-6.25	SLA-3*04:06|361	-0.2
SLA-2*04:01|364	0	SLA-3*03:06|361	-31.3
SLA-2*05:07|364	-26.2	SLA-3*06:02|361	-18.1
SLA-2*06:09|364	-34.3	SLA-3*06:01|361	-16.8
SLA-2*10:09|364	-35.3	SLA-3*04:01|361	0
SLA-2*08:05|364	-36.2	SLA-3*03:13|361	-33.1
SLA-2*10:04|364	-33.2	SLA-3*03:02|361	-28.65
SLA-2*02:01|364	-39.4	SLA-3*05:03:01|361	-18.15
SLA-2*07:01|364	-34.6	SLA-3*01:01|361	-17.9
SLA-2*16:02|364	-33.3	SLA-3*05:08|361	-18.9
SLA-2*13:03|364	-26.1	SLA-3*04:05|361	-0.3
SLA-2*06:01|364	-37	SLA-3*04:04|361	-21.55
SLA-2*16:01|364	-30.6	SLA-3*04:08|361	-18.1
SLA-2*11:05|364	-28.1	SLA-3*06:03|361	-21.5
SLA-2*11:01:02|364	-25	SLA-3*03:04|361	-24
SLA-2*11:02|364	-27.7		
SLA-2*01:02|364	-20.7		
SLA-2*07:04|364	-24		
SLA-2*13:01|364	-25.2		
SLA-2*12:02|364	-31.5		
SLA-2*08:01|364	-28.3		
SLA-2*10:01|364	-31.6		
SLA-2*15:01|364	-25.1		
SLA-2*03:03|364	-29.2		
SLA-2*06:07|364	-34.9		

SLA-2*06:05|364	-34.8		
SLA-2*01:03|364	-20.7		
SLA-2*06:02:02|364	-35.5		
SLA-2*05:02|364	-30.1		
SLA-2*06:02:01|364	-35.5		
SLA-2*04:02:02|364	-5.6		
SLA-2*02:02|364	-41.1		
SLA-2*04:02:01|364	-5.6		
SLA-2*03:01|364	-40.4		
SLA-2*16:03|364	-32.5		
SLA-2*05:09|364	-29.3		
SLA-2*04:03|364	-11.9		
SLA-2*07:03|364	-31.2		
SLA-2*08:07|364	-32.1		
SLA-2*12:01|364	-30.5		
SLA-2*08:03|364	-31.9		
SLA-2*06:06|364	-37.8		
SLA-2*06:04|364	-40.3		
SLA-2*10:05|364	-33.9		
SLA-2*01:01|364	-19.7		
SLA-2*11:03|364	-27.6		
SLA-2*14:01|364	-32.2		
SLA-2*07:06|364	-33.6		
SLA-2*08:04|364	-30.9		
SLA-2*11:04|364	-28.4		
SLA-2*08:06|364	-32.3		
SLA-2*05:04|364	-33		
SLA-2*04:04|364	-12.3		
SLA-2*06:16|364	-39.6		

Supplementary Table 1: **The CAAI scores for SLA -2 and SLA-3**. The CAAI scores for SLA -2 and SLA-3 were calculated using the weighed amino acid scoring function based on our developed algorithm as observed in Table 1. [a]The sequences of all SLA alleles in these studies are obtained from Immunopolymorphism Database (https://www.ebi.ac.uk/ipd/licence/) and that database classifies each protein as SLA-2 or SLA-3.

SLA Alleles	Docking Score	CAAI Rank	Sequence Similarity		SLA Alleles	Docking Score	CAAI Rank	Sequence Similarity
SLA06108|SLA-1*04:04|361	-122.3	1	1		SLA06120|SLA-1*08:08|361	-83.3	45	49
SLA06109|SLA-1*04:05|361	-112.5	2	2		SLA06118|SLA-1*08:13|361	-91.1	41	50
SLA06132|SLA-1*13:02|361	-106.3	3	3		SLA06142|SLA-1*10:02|361	-108	62	51
SLA06108|SLA-1*04:04|361	-122.3	1	1		SLA09716|SLA-1*21:03|361	-101.9	38	52
SLA06107|SLA-1*04:03|361	-123.9	4	6		SLA09737|SLA-1*11:11|361	-101.6	59	57
SLA08460|SLA-1*17:02|361	-100.8	6	7		SLA08459|SLA-1*16:03|361	-104.4	57	58
SLA06112|SLA-1*06:02|361	-93.9	7	8		SLA06125|SLA-1*11:03|361	-90.7	63	59
SLA06111|SLA-1*06:01|361	-92.3	9	9		SLA06143|SLA-1*10:01|361	-79.8	65	60
SLA06135|SLA-1*17:01|361	-97.2	11	10					
SLA09742|SLA-1*14:04|361	-102.4	44	39		SLA08452|SLA-1*11:07|361	-83.9	66	62
					SLA08447|SLA-1*08:11|361	-96.9	61	63
SLA08442|SLA-1*07:06|361	-96.1	43	41					
SLA06138|SLA-1*07:03|361	-103.7	16	19		SLA08461|SLA-1*19:01|361	-98.1	64	64
					SLA08453|SLA-1*11:08|361	-74.9	68	66
SLA06129|SLA-1*12:03|361	-97.9	29	20					
SLA06128|SLA-1*12:01|361	-104.6	30	21		SLA08453|SLA-1*11:08|361	-74.9	68	66
					SLA08445|SLA-1*08:09|361	-93.9	58	67
SLA06115|SLA-1*08:01|361	-105.7	20	24		SLA06124|SLA-1*11:02|361	-109.6	67	68
SLA09741|SLA-1*08:18|361	-80.5	37	45		SLA08455|SLA-1*11:10|361	-104.8	69	69
SLA08446|SLA-1*08:10|361	-96.3	36	47					
SLA06121|SLA-1*08:03|361	-91.9	46	48					

Supplementary Table 2: Docking of different sets of SLA1 with the referenced crystalized epitope obtained from PDB ID: 3QQ3. The sequences of all SLA alleles are obtained from Immunopolymorphism Database (https://www.ebi.ac.uk/ipd/licence/). The CAAI scores were calculated based on our developed algorithm. We performed a docking analysis of the reference SLA-1* 04:01 allele's crystalized epitope to the random SLA-1 allele using HADDOCK software. If the CAAI or sequence similarity ranked score shows agreement with the docking score (i.e. the top-ranked allele based on the CAAI method has a more negative docking score between the crystalized epitope and the SLA), the ranking is coloured green. If the CAAI or sequence similarity ranking score and the docking score are not in agreement, the rankings are colored red. Results showed that 14 out of the 17 randomly selected pairs show agreement

between the CAAI ranking and the docking scores, and the sequence similarity scoring show agreement with the docking score 11 out of 17 times.

SLA1 Alleles	Pairwise distance to	Pairwise Distance Rank	CAAI Rank

	SLA-1*0401		
SLA-1*04:04	0.00555557	1	1
SLA-1*04:05	0.008344972	2	2
SLA-1*13:02	0.022409901	3	3
SLA-1*04:03	0.025246783	4	4
SLA-1*13:01	0.025246783	7	5
SLA-1*17:02	0.028091735	8	6
SLA-1*06:02	0.030944804	5	7
SLA-1*04:02	0.025246783	6	8
SLA-1*06:01	0.030944804	9	9
SLA-1*02:01	0.054067221	14	10
SLA-1*17:01	0.045333541	13	11
SLA-1*15:02	0.048236301	10	12
SLA-1*02:02	0.054067221	12	13
SLA-1*15:01	0.051147511	11	14
SLA-1*02:03	0.056995481	15	15
SLA-1*07:03	0.068795028	16	16
SLA-1*01:01	0.065832063	17	17
SLA-1*07:02	0.065832063	18	18
SLA-1*07:01	0.068795028	19	19
SLA-1*08:01	0.080735468	30	20
SLA-1*08:02	0.080735468	23	21
SLA-1*08:06	0.080735468	33	22
SLA-1*08:12	0.086759583	44	23
SLA-1*09:01	0.077736965	37	24
SLA-1*08:14	0.083742989	21	25
SLA-1*09:02	0.074747427	27	26
SLA-1*07:05	0.083742989	35	27
SLA-1*12:02	0.080735468	22	28

SLA-1*12:03	0.074747427	25	29
SLA-1*12:01	0.074747427	24	30
SLA-1*08:07	0.083742989	20	31
SLA-1*07:04	0.089785304	51	32
SLA-1*14:02	0.086759583	32	33
SLA-1*11:04	0.083742989	29	34
SLA-1*18:01	0.083742989	38	35
SLA-1*08:10	0.098917787	42	36
SLA-1*08:18	0.09586435	39	37
SLA-1*21:03	0.101980577	45	38
SLA-1*14:03	0.086759583	47	39
SLA-1*08:15	0.09586435	41	40
SLA-1*08:13	0.101980577	56	41
SLA-1*15:03	0.086759583	31	42
SLA-1*07:06	0.092820208	50	43
SLA-1*14:04	0.089785304	36	44
SLA-1*08:08	0.098917787	46	45
SLA-1*08:03	0.098917787	28	46
SLA-1*14:05	0.09586435	34	47
SLA-1*11:09	0.083742989	26	48
SLA-1*14:01	0.09586435	48	49
SLA-1*08:05	0.105052776	53	50
SLA-1*20:01	0.09586435	49	51
SLA-1*11:06	0.092820208	43	52
SLA-1*21:01	0.111225635	52	53
SLA-1*11:01:01	0.105052776	55	54
SLA-1*11:01:02	0.105052776	54	55
SLA-1*11:05	0.101980577	62	56
SLA-1*16:03	0.108134443	57	57

**SLA-1*08:09**	**0.120556963**	**66**	**58**
**SLA-1*11:11**	**0.105052776**	**59**	**59**
**SLA-1*08:04**	**0.114326413**	**60**	**60**
**SLA-1*08:11**	**0.111225635**	**58**	**61**
**SLA-1*10:02**	**0.101980577**	**40**	**62**
**SLA-1*11:03**	**0.108134443**	**68**	**63**
**SLA-1*19:01**	**0.114326413**	**64**	**64**
**SLA-1*10:01**	**0.108134443**	**65**	**65**
**SLA-1*11:07**	**0.111225635**	**63**	**66**
**SLA-1*11:02**	**0.120556963**	**67**	**67**
**SLA-1*11:08**	**0.117436835**	**61**	**68**
**SLA-1*11:10**	**0.123686856**	**69**	**69**
**SLA-1*23:01**	**0.123686856**	**70**	**70**

Supplementary Table 3: **The pairwise distance score**. The pairwise distances scores were calculated using Omega software. The distance scores were evaluated for 70 SLA-1 alleles, but only data for each allele related to SLA-1*04:01 are shown here and compared to CAAI ranking**.** Pairwise distance scoring is a commonly used approach to compare sequence similarity.

a

b

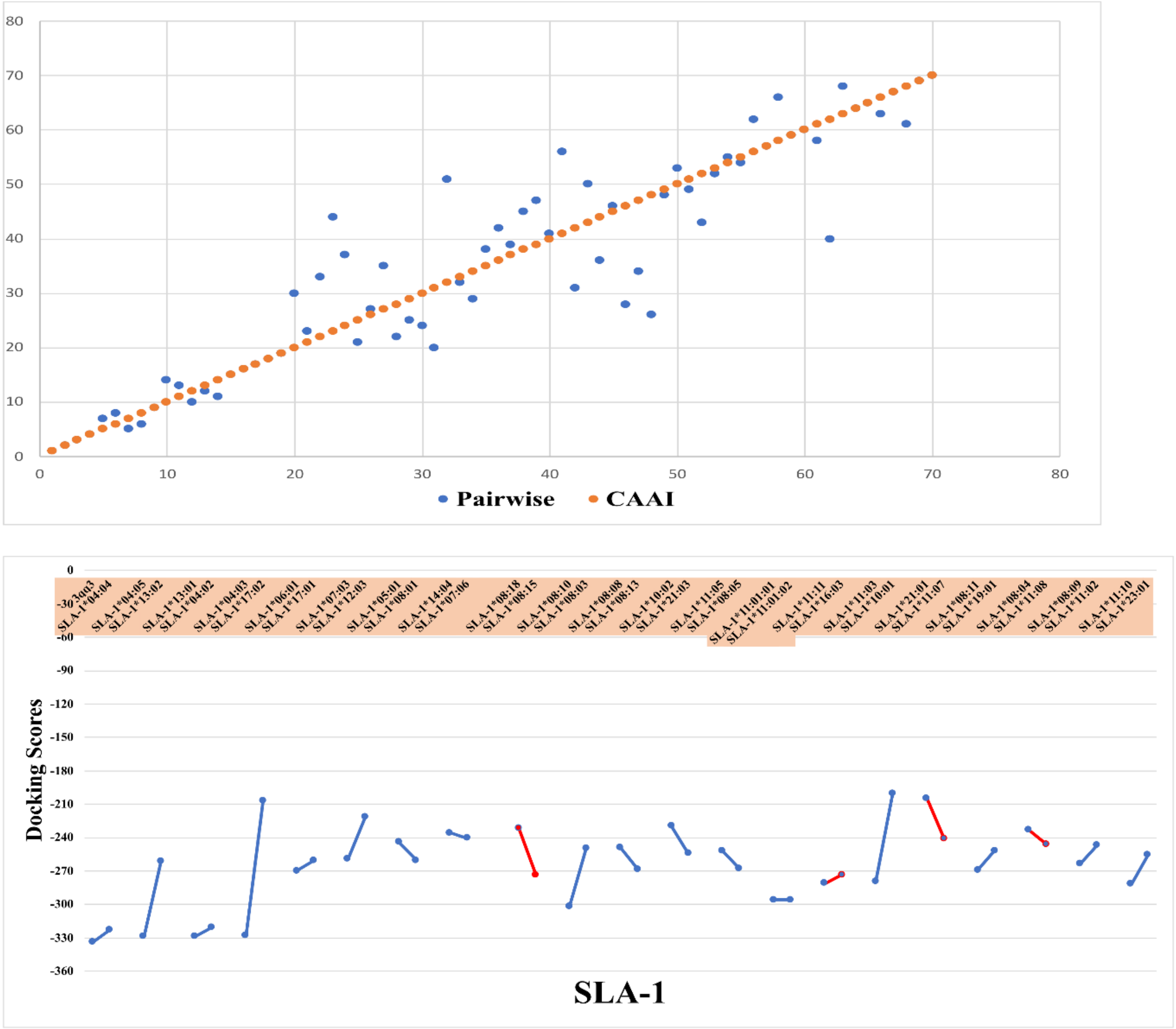

Supplementary Figure 6: Correlation coefficient and results of blind docking. (a) Spearman's correlation coefficient (r) value and scatter plot of SLA-1 rank vs. pairwise distance ranking. The estimated coefficient is r = 0.9311 (**b**) the 21 random SLA-1 sets were selected, and the crystalized 3QQ3 epitope was docked into their peptide binding sites. The red lines are not in agreement with CAAI, and the blue lines are predicted accurately through the CAAI ranking.

Position	MHC	Peptide	Score_EL	%Rank_EL	Score_BA	%Rank_BA	Aff(nM)	BindLevel
12	SLA-1:0101	STAPQKVLL	0.3476220	0.244	0.192816	1.216	6207.74	SB
126	SLA-1:0101	AANDNHAFV	0.3216570	0.313	0.292388	0.123	2113.72	SB
127	SLA-1:0101	ANDNHAFVV	0.3057150	0.368	0.293043	0.121	2098.79	SB
24	SLA-1:0201	ITYTPVMIY	0.7777670	0.022	0.455063	0.056	363.62	SB
18	SLA-1:0201	VLLAFSITY	0.6326360	0.126	0.409061	0.170	598.14	SB

84	SLA-1:0201	GVYSAIETW	0.5805880	0.204	0.335730	0.635	1322.47	SB
86	SLA-1:0201	YSAIETWKF	0.5307780	0.303	0.371815	0.339	895.00	SB
67	SLA-1:0201	STNRVALTM	0.4907460	0.407	0.365261	0.379	960.77	SB
24	SLA-1:0202	ITYTPVMIY	0.7777670	0.022	0.455063	0.056	363.62	SB
18	SLA-1:0202	VLLAFSITY	0.6326360	0.126	0.409061	0.170	598.14	SB
84	SLA-1:0202	GVYSAIETW	0.5805880	0.204	0.335730	0.635	1322.47	SB
86	SLA-1:0202	YSAIETWKF	0.5307780	0.303	0.371815	0.339	895.00	SB
67	SLA-1:0202	STNRVALTM	0.4907460	0.407	0.365261	0.379	960.77	SB
24	SLA-1:0401	ITYTPVMIY	0.6799820	0.399	0.387275	0.768	757.14	SB
67	SLA-1:0401	STNRVALTM	0.6796060	0.400	0.490134	0.280	248.80	SB
84	SLA-1:0501	GVYSAIETW	0.7809900	0.027	0.385499	0.122	771.83	SB
24	SLA-1:0501	ITYTPVMIY	0.6234010	0.208	0.270152	0.753	2688.65	SB
75	SLA-1:0501	MGAVVALLW	0.6118910	0.231	0.486082	0.010	259.95	SB
84	SLA-1:0601	GVYSAIETW	0.4372150	0.226	0.284027	0.433	2313.85	SB
86	SLA-1:0601	YSAIETWKF	0.4200690	0.261	0.317164	0.214	1616.69	SB
125	SLA-1:0601	IAANDNHAF	0.3582800	0.398	0.251025	0.821	3306.83	SB
12	SLA-1:0601	STAPQKVLL	0.3516070	0.420	0.189578	2.494	6429.08	SB
67	SLA-1:0601	STNRVALTM	0.3335260	0.484	0.285078	0.423	2287.69	SB
24	SLA-1:0701	ITYTPVMIY	0.8006910	0.052	0.583306	0.079	90.79	SB
14	SLA-1:0701	APQKVLLAF	0.5946270	0.183	0.338724	0.811	1280.31	SB
18	SLA-1:0701	VLLAFSITY	0.4530070	0.371	0.480679	0.243	275.60	SB
24	SLA-1:0702	ITYTPVMIY	0.8006910	0.052	0.583306	0.079	90.79	SB
14	SLA-1:0702	APQKVLLAF	0.5946270	0.183	0.338724	0.811	1280.31	SB
18	SLA-1:0702	VLLAFSITY	0.4530070	0.371	0.480679	0.243	275.60	SB
24	SLA-1:0801	ITYTPVMIY	0.6399790	0.062	0.416291	0.128	553.13	SB
18	SLA-1:0801	VLLAFSITY	0.5384150	0.121	0.403104	0.148	637.96	SB
84	SLA-1:0801	GVYSAIETW	0.3295280	0.463	0.238255	1.063	3796.79	SB
12	SLA-1:1101	STAPQKVLL	0.1688150	0.139	0.174854	3.316	7539.39	SB
148	SLA-1:1101	VPGLKSLVL	0.1275950	0.305	0.196791	2.030	5946.41	SB
161	SLA-1:1201	AVKQGVVNL	0.5928010	0.065	0.281172	4.084	2386.44	SB
12	SLA-1:1201	STAPQKVLL	0.5718930	0.088	0.322626	1.909	1523.91	SB
24	SLA-1:1201	ITYTPVMIY	0.5421200	0.134	0.338876	1.368	1278.21	SB
146	SLA-1:1201	TLVPGLKSL	0.5088140	0.193	0.292912	3.327	2101.77	SB
84	SLA-1:1201	GVYSAIETW	0.4302300	0.455	0.284101	3.885	2312.00	SB
40	SLA-1:1201	RLLGLLHLL	0.4300760	0.456	0.363299	0.806	981.38	SB
84	SLA-1:1301	GVYSAIETW	0.4916060	0.272	0.316679	0.521	1625.19	SB
86	SLA-1:1301	YSAIETWKF	0.4527820	0.358	0.354559	0.247	1078.72	SB
125	SLA-1:1301	IAANDNHAF	0.4203380	0.458	0.291214	0.763	2140.74	SB

Supplementary Table 4: **Strong binders to 12 SLA-1 by NetMHCpan**. Listing of the top PRRSV M protein epitopes that bind strongly to the 12 SLA-1 as determined by NETMHCpan4.1 tool. Position refers to the position in the PRRSV M protein of the first of the nine amino acids in the epitope. Different colors are used to differentiate the SLA-1 alleles. Selection of these epitopes as strong binding epitopes (SB) to the indicated SLA allele is based on % El_rank according to the default criteria in the NETMHCpan4.1 software.
